# Experimental determination of effective X-ray attenuation coefficients of 3D-printed materials under clinical mammography spectra

**DOI:** 10.3389/fbioe.2025.1719551

**Published:** 2026-02-05

**Authors:** Adrián Belarra, Irene Hernández-Girón, Julia Garayoa, Luis Carlos Martínez, Julio Valverde, María José Rot, Alejandro Ferrando, Antonio Martín, Margarita Chevalier

**Affiliations:** 1 Medical Physics, Radiology and Rehabilitation Department, Facultad de Medicina, Universidad Complutense de Madrid, Madrid, Spain; 2 School of Physics, University College Dublin (UCD), Dublin, Ireland; 3 Servicio de Protección Radiológica, Hospital Universitario Fundación Jiménez Díaz, Madrid, Spain; 4 Servicio de Radiofísica Hospitalaria, Hospital Universitario 12 de Octubre, Madrid, Spain; 5 Unidad de Tecnologías Avanzadas en Diseño e Impresión 3D (UTADI 3D), Instituto i+12. Hospital Universitario 12 de Octubre, Madrid, Spain

**Keywords:** effective attenuation coefficient, 3D printing, beam hardening, scattered radiation, X-ray breast imaging, polyenergetic beams

## Abstract

**Background:**

3D printing enables the fabrication of customized breast phantoms for image quality assessment in digital mammography (DM) and digital breast tomosynthesis (DBT). A major challenge is the absence of standardized, accessible methods to characterize the attenuation properties of 3D-printed materials under clinical DM/DBT spectra.

**Methods:**

An experimental framework was implemented to determine the effective X-ray attenuation coefficient (*μ*_*eff*_) of six 3D-printed polymers (PLA, PET, resin, ABS, ABS+, HIPS) and reference breast tissue-equivalent materials (CIRS plates simulating different breast glandular/adipose ratios (BR) and PMMA) using two commercial DM/DBT systems, with and without anti-scatter grid. Step-wedges (0.5–5.5 cm) were imaged across multiple kVp and filter settings. The *μ*_*eff*_ were obtained from measurements on images and fitted to an empirical model yielding *μ*_*0*_ (attenuation at thickness tending to zero) and *k* (decay rate) to characterize beam hardening and scatter influences. 3D-reference material equivalences were evaluated based on *μ*_*eff*_ and *μ*_*0*_ *.*

**Results:**

Beam hardening and scatter reduced *μ*_*eff*_ with thickness, by 6%–14% with grid and 12%–28% without grid, with scatter contributing 47%–76% of the reduction in no-grid acquisitions. No significant differences were observed between the two mammography systems. Based on *μ*_*eff*_ values, attenuation equivalences (within ±6%) were identified between 3D-printed and reference breast tissue-equivalent materials: PLA with BR 100/0; PET and resin with BR 70/30 and PMMA; ABS+ with BR 30/70 and BR 50/50. ABS and HIPS showed larger mismatches. The empirical model achieved excellent fits (R^2^ > 0.99), with *μ*_*0*_ values preserving attenuation ranking and enabling derivation of equivalent glandular proportions.

**Conclusion:**

This framework demonstrates that routine clinical mammography systems can be used directly, without specialized instrumentation, to characterize 3D-printed materials as tissue surrogates. Several low-cost, widely available polymers were shown to reproduce breast tissue attenuation, supporting the local fabrication of anthropomorphic breast phantoms for realistic and clinically relevant image quality evaluation.

## 1 Introduction

The evaluation of image quality in medical imaging systems is essential for accurate diagnosis from resulting images. This task has become increasingly complex due to the growing capability of these systems to generate multimodal and multiparametric data, involving vendor-specific image acquisition and processing methods. This is particularly relevant in breast imaging, where X-ray based modalities such as digital mammography (DM), digital breast tomosynthesis (DBT), contrast-enhanced mammography (CEM), and breast computed tomography (bCT) have been introduced in recent years.

Breast X-ray imaging systems aim to optimize soft-tissue contrast to improve the detectability of subtle lesions. During both DM and DBT examinations, the breast is compressed to reduce its thickness, which lowers patient dose, minimizes scattered radiation, and improves image sharpness. DM provides a single two-dimensional projection of the compressed breast, but its diagnostic performance may be limited by tissue superposition, which can obscure relevant findings. DBT was developed to address this limitation by acquiring multiple low-dose projections over a limited angular range and reconstructing them into a three-dimensional dataset. This approach reduces the impact of overlapping structures and improves lesion conspicuity, particularly for masses and architectural distortions.

The X-ray tube typically employs molybdenum or tungsten anodes in combination with dedicated filters (Mo, Rh, Ag, Al) to shape the beam spectrum, operating at relatively low tube voltages (25–35 kVp). To reduce scattered radiation and improve image quality, an anti-scatter grid is generally used in DM acquisitions, whereas in DBT the grid is removed due to the need for multiple low-dose projections (Sechopoulos, 2013). Image detection in DM and DBT can be achieved through indirect detectors, which convert X-rays into light and subsequently into electronic signals, or direct detectors, which convert X-rays directly into charge.

DM and DBT play a pivotal role in early breast cancer detection within screening programs worldwide. The success of these programs relies on accurate image quality evaluation, preferably under conditions close to clinical practice. Conventional phantoms for image quality evaluation typically consist of test objects embedded within homogeneous backgrounds, allowing assessment of system imaging parameters such as resolution, noise, contrast resolution and stability (EFOMP, 2015; EFOMP, 2025). However, clinical breast images exhibit complex, heterogenous backgrounds due to the uneven distribution of breast glandular and adipose tissues, which can hinder lesion detection.

To address this limitation in conventional phantoms, digital anthropomorphic breast phantoms aim to replicate the three-dimensional breast tissue distribution and image contrasts to assess the imaging system’s ability to detect lesions in complex backgrounds (Bliznakova, 2020; Sarno et al., 2024). In recent years, 3D printing has emerged as a promising approach for producing realistic physical anthropomorphic phantoms that closely replicate the anatomy of organs such as lung, liver, chest, and breast (Bliznakova, 2020; Sarno et al., 2024; Flippou and Tsoumpas, 2018; Zhang et al., 2019; Hernandez-Giron et al., 2019; Okkalidis, 2022; Dukov et al., 2022). The growing accessibility of 3D printers, particularly fused deposition modeling (FDM) and stereolithography (SLA) modalities, has driven their use due to their cost-effectiveness and local manufacturing capabilities, making them attractive for medical physics applications like image quality assessment. Various 3D printing technologies have been explored for breast phantom fabrication, including FDM (Varallo et al., 2022), SLA (Malliori et al., 2020; Schopphoven et al., 2019), selective laser sintering (SLS) (Mainprize et al., 2018), photopolymer inkjet (Carton et al., 2011; Kiarashi et al., 2015) and inkjet printing with doped inks (Ikejimba et al., 2017). Among these, FDM and SLA provide the widest range of materials, and they are currently the most widely adopted for anthropomorphic phantom construction due to their cost-effectiveness.

To produce an anthropomorphic breast phantom suitable for image quality evaluation, the 3D printing materials must replicate the radiological properties of the tissues they are intended to mimic. Attenuation properties of materials such as photopolymers for SLA and photopolymer inkjet, thermoplastics for FDM (i.e., PLA, Nylon, PET-G, ABS) and other materials such as paraffin and gelatine have been studied using CT (Dancewicz et al., 2017; Ma et al., 2021; Kozee et al., 2023) or standardized X-ray qualities (from Radiation Quality Reference (RQR) 3 to RQR 10) (International Electrotechnical Commission (IEC), 2025; Veneziani et al., 2016; Kunert et al., 2022; Savi et al., 2021; Villani et al., 2021). However, these X-ray beam qualities exhibit a spectrum with higher energies than those typically used in DM and DBT. Other study (Oliveira et al., 2022) employed the standard beam qualities for mammography RQR 2-M (Mo/Mo 28 kVp) and RQR 4-M (Mo/Mo 35 kVp) with mean energies of 15.4 and 16.3 keV, respectively. Attenuation coefficients in the 14–60 keV range have been reported in investigations using synchrotron radiation (Ivanov et al., 2018; Mettivier et al., 2022). However, these studies relied on highly specialized instrumentation and facilities that are not readily accessible.

Furthermore, the X-ray attenuation of 3D printing materials can vary substantially even when they share the same base composition, due to the presence of additives introduced to modify color or thermo-mechanical properties (Ma et al., 2021; Mettivier et al., 2022). Therefore, experimental characterization of the attenuation of each selected material is mandatory. Such variability highlights the need to evaluate the attenuation properties of the exact material batch to be used—whether a filament spool or a resin container—even when sourced from the same manufacturer. For this reason, it becomes essential to develop an experimental methodology to characterize attenuation under clinically relevant conditions, using accessible procedures that can be implemented directly at the site of phantom fabrication, such as a hospital.

In this study, the effective attenuation coefficients (*μ*_*eff*_) of several 3D-printed materials were experimentally determined using multiple clinical spectra from two different vendor systems of DM and DBT. In the systems considered here, direct conversion technology based on amorphous selenium (a-Se) is employed. To obtain *μ*_*eff*_ values for different thicknesses within a single acquisition, a step-wedge model was employed. The suitability of the 3D-printed materials to mimic breast tissue attenuation was assessed by comparing their *μ*_*eff*_ values with those of CIRS plates (Sun Nuclear, Melbourne, FL, United States) (CIRS–Sun Nuclear, 2025), which are widely recognized as breast tissue-equivalent standards (Byng et al., 1998; Poletti et al., 2002; Geraldelli et al., 2013; Heine and Behera, 2006; Heine and Thomas, 2008). Polymethyl methacrylate (PMMA) was also included, given its routine use in mammography quality control. Furthermore, because polyenergetic spectra were used, the effects of scattered radiation and beam hardening on *μ*_*eff*_ were analyzed by fitting the results to a two-parameter empirical model dependent on thickness. This approach aims to provide a straightforward characterization of attenuation properties under polyenergetic X-ray beams through the two parameters derived for two different vendor DM/DBT systems.

## 2 Materials and equipment

### 2.1 3D-printed and reference materials

A selection of 3D-printed materials (3D materials hereinafter) was investigated due to their wide availability and compatibility with low- and mid-range FDM and SLA printers suitable for local use. 3D materials with same base composition have also been employed in 3D-printed anthropomorphic breast phantoms for mammography, target modality of this study (Schopphoven et al., 2019; Varallo et al., 2022; Malliori et al., 2020; Mainprize et al., 2018). They included polylactic acid (PLA; grey, ρ = 1.24 g/cm^3^) (Crystal Silver PLA, 2025), high impact polystyrene (HIPS; white, ρ = 1.04 g/cm^3^) (Natural, HIPS, 2025), polyethylene terephthalate (PET; grey, ρ = 1.28 g/cm^3^) (PET grey, 2025), resin (resin; clear, ρ = 1.15–1.20 g/cm^3^) (Formlabs, 2025) and acrylonitrile butadiene styrene (ABS; uncolored, ρ = 1.04 g/cm^3^ and ABS+; white, ρ = 1.06 g/cm^3^) (Natural ABS, 2025; White ABS, 2025). ABS+ refers to vendor-modified ABS with undisclosed additives that typically reduce shrinkage/warping and improve bed adhesion relative to standard ABS.

**FIGURE 1:**
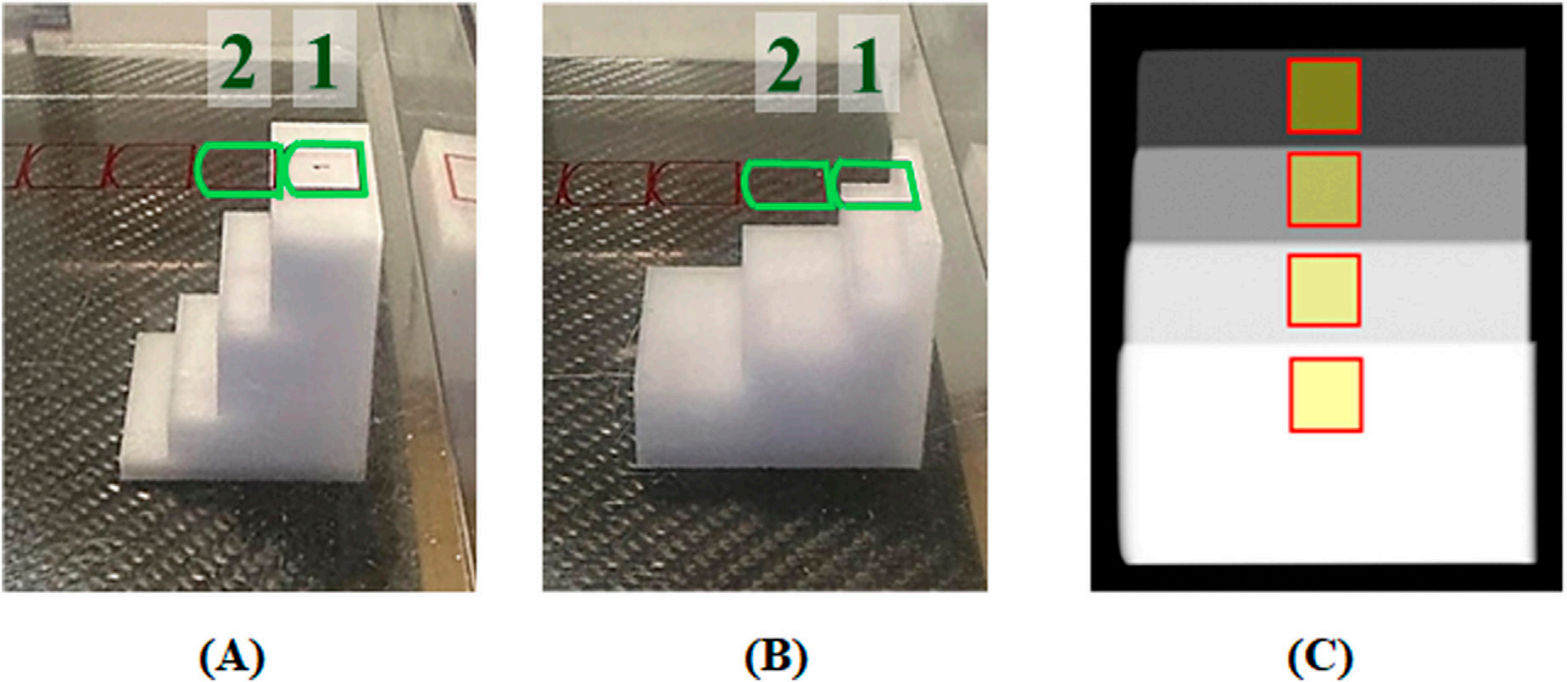
Step-wedge positioned on the breast-support table at **(A)** first and **(B)** second positions. The second position is the result of applying a 90°-flip to the step-wedge. The first position was used to acquire thicknesses of 0.5, 1.5, 3.5 and 5.5 cm and the second, 2.0, 2.5 and 4.5 cm. Numbered boxes (added in green to ensure visibility) in **(A,B)** indicate AEC sensor locations; position one and two were used in this study **(C)** Cropped DM image of the step-wedge in the first position with yellow ROIs (1.0 × 0.9 cm^2^) used for measurements of pixel values.

A step-wedge model was used for experimental measurements (see Figure 1), enabling acquisition of multiple thicknesses within a single X-ray exposure. For 3D materials, step-wedges were designed in Tinkercad (2025) with steps of 0.5, 1.5, 3.5 and 5.5 cm, offset by 1.0 cm. Samples were printed using two FDM printers: BCN3D Sigma R19 (BCN3D; Barcelona, Spain) and Bambu X1C (Bambu Lab; Shenzhen, China) and a SLS printer: Form 3BL (Formlabs, 2025; Somerville, Massachusetts, United States).

Printing parameters (Table 1) were derived from prior optimization tests to maximize attenuation uniformity and minimize imaging artifacts (Belarra et al., 2023). For FDM printers, top/bottom solid layers and perimeters (walls) were disabled so the step-wedge was printed as a single continuous infill, achieving uniform density and attenuation. Infill patterns and near-solid densities were selected accordingly—99% on the Bambu Lab X1 Carbon (printer-imposed limit) and 100% on the BCN3D Sigma R19—and nozzle diameters were chosen per material following manufacturers’ specifications to reduce printing issues. Additionally, the line width was set slightly larger than the nozzle diameter to increase infill line overlap, reducing internal voids and further improving attenuation uniformity. All samples were visually inspected for surface printing defects. The input files for the printer with all the printing parameters are available for requesting to the corresponding author.

**TABLE 1:** Printing parameter settings for each 3D printer and material. Not specified parameters were set according to manufacturer recommendation for the corresponding printer and filament.

Printer	Material	Printing temperature (°C)	Nozzle diameter (mm)	Layer height (mm)	Line width (mm)	Infill (type, %)	Speed (mm/s)
BCN3D Sigma	PLA	220	0.3	0.14	0.32	Gyroid, 100	70
	ABS, HIPS	260	0.4	0.14	0.42		
Bambu X1C	PET, ABS+	250	0.2	0.14	0.22	Lines, 99	
Form 3BL	Resin	—	0.085*	0.1	—	—	—

*This value corresponds to screen resolution of the SLA printer.

As a benchmark, commercially available breast tissue-equivalent materials (reference materials hereinafter) were selected to determine the equivalence of 3D materials and breast tissues. Resin plates (10 × 12 cm^2^) from CIRS (CIRS–Sun Nuclear, 2025) were used with compositions equivalent to the following percentages of glandular (first value) and adipose (second value) tissues: BR 0/100, BR 30/70, BR 50/50, BR 70/30, BR 100/0 (Hammerstein et al., 1979). For each composition, these plates were stacked to form two step-wedges of 0.5, 1.5, 3.5, 5.5 cm and 1.0, 2.0, 2.5, 4.5 cm. Semicircular PMMA plates (Leeds Test Objects, York, United Kingdom (Leeds Test Objects, 2025)) with a diameter of 24 cm and measured density of 1.180 ± 0.002 g/cm^3^ were also considered. PMMA plates were stacked to get a step-wedge with thicknesses of 0.5, 1.5, 2.5, 3.5, 4.5 and 5.5 cm. Thicknesses of all steps (reference and 3D materials) were verified using a caliper (0.05 mm resolution).

### 2.2 X-ray image systems and experimental setup

The step-wedges (reference and 3D) were imaged in two clinical breast X-ray imaging systems: Hologic 3Dimensions (Hologic Inc., Marlborough, Massachusetts, United States) (hereinafter, M1) and Siemens Mammomat Inspiration (Siemens Healthineers AG, Forchheim, Germany) (hereinafter, M2). Both systems support DM and DBT, employing tungsten (W) anode X-ray tubes. For DM, M1 selects 50 µm rhodium (Rh) or silver (Ag) filters, while M2 only uses 50 µm Rh. For DBT, M2 uses the same filtration as in DM (50 µm Rh), while M1 uses 700 µm aluminum (Al). Both systems are equipped with a-Se detectors.

For M1, exposure factors (filtration and kVp) were selected based on the most frequent clinical settings for DM and DBT of 4-6 cm thick breasts. These were obtained by inspection of the DICOM data stored in the patient images. Selected kVp were 27, 29, 31 for DM (W/Rh, W/Ag) and DBT (W/Al). For M2, kVp values were obtained from AEC acquisitions on varying PMMA thicknesses for both DM and DBT (W/Rh). Step-wedges of BR 0/100, BR 50/50, BR 100/0, PLA, ABS and HIPS were imaged on M2.

## 3 Methods

### 3.1 Theoretical framework

For narrow-beam monoenergetic X-rays, Beer-Lambert’s law describes the relationship between the incident and the transmitted energy fluence through a homogeneous material of thickness *t* and linear X-ray attenuation coefficient *μ*. In a mammography system, however, a divergent and polyenergetic X-ray beam is used. The transmitted intensity is usually registered with a flat-panel detector. The pixel values of this type of detector, after subtracting darkfield image (*DF*), are directly proportional to the total energy absorbed by the detector surface. Furthermore, the pixel values are affected by the X-ray beam divergence, scatter radiation generated within the medium, and beam hardening due to the polyenergetic spectrum. The effective X-ray attenuation coefficient (*µ*_*eff*_) as a function of thickness is defined as:

$$\mu_{eff}\left(t\right)=\frac{1}{t}\ln\left(\frac{PV\left(0\right)-DF}{PV\left(t\right)-DF}\right)$$ (1)

where *PV(t)* and *PV(0)* denote the detector pixel values with and without a homogeneous medium of thickness *t* placed on the detector, respectively.

The presence of scatter radiation influences the signal values provided by the detector and in consequence invalidates the use of Equation 1 to determine the actual value of the attenuation coefficient. Therefore, the *µ*_*eff*_ values are for comparative purposes only, assuming similar scatter-to-primary ratios for materials with similar composition and thickness when irradiated under identical conditions; thus, scatter behaves as a common bias and has limited impact on pairwise comparisons of *µ*_*eff*_. In addition, the impact of the major presence of scatter in *µ*_*eff*_ values is analyzed by removing the anti-scatter grid during image acquisition.

### 3.2 Experimental procedure

For DM and DBT acquisitions, step-wedges were positioned with their thickest step centered on the side where X-ray beam fluence is highest and the thickest breast region (nearest to the chest wall) is positioned in the clinical scenario (see Figures 1A,B). The compression paddle remained in contact with the step-wedges. For M1, two step-wedge positions of the 3D step-wedges were considered to have three additional thicknesses (2.0, 2.5, 4.5 cm) as shown in Figures 1A,B.

For both DM and DBT acquisition, the appropriate mAs for each kVp/filtration–material combination was first determined with AEC, placing the sensor under the thickest (or next-thickest, depending on material attenuation) step (Figures 1A,B). AEC was then disabled, and the mAs was set manually to the console value closest to the AEC-derived estimate. For DM, three images were acquired per exposure setting with the anti-scatter grid in place. To assess the effect of scatter on *μ*_*eff*_ *(t)*, additional DM images on system M1 were acquired without (w/o) the anti-scatter grid, using the same exposure factors as with the grid. As required, DBT was performed without the grid (Sechopoulos, 2013). For both systems, three DBT series were acquired with the X-ray tube fixed at 0° (quality-control menu in the mammography system), and from each series only the central projection was selected for analysis. Three dark-field images (*DF* in Equation 1) were obtained by placing a 2 mm lead sheet over the detector with the minimum kVp/mAs settings. For each exposure condition, three images without the homogeneous medium (*PV(0)* in Equation 1) were acquired after removing the step-wedges. Hereinafter, *PV(0)* is denoted as flat-field image.

All for-processing images were dark-field corrected, averaged and logarithmically transformed, according to Equation 1. Mean pixel value and standard deviations were measured in a set of 1.0 × 0.9 cm^2^ ROIs centered on each step (Figure 1C). The exact position of each ROI was determined based on the uniformity of a line profile across each step. For each thickness, the effective X-ray attenuation coefficient *μ*_*eff*_ *(t)* was computed from the measured mean pixel value and measured thickness (Equation 1). Uncertainties were estimated via error propagation.

Thus, beam-divergence effects are minimized by ROI placement (detector midline, near the chest-wall edge) and division by flat-field images (see Equation 1). It should be noted that the maximum effective thickness traversed by the X-ray beam through the step-wedge differs by only 0.4% from its nominal thickness.

To assess 3D printing repeatability, five 2 × 2 × 2 cm^3^ cubes were printed in a single run using ABS+ (Bambu X1C) and uncolored ABS (BCN3D Sigma R19). Thicknesses were measured using a caliper with a 0.05 mm uncertainty. Each cube was individually imaged using a micro-focus X-ray radiography setup (Hamamatsu L10951-04 and 50 μm pixel size flat panel Hamamatsu C7940DK-02) at 50 kVp and 100 μAs. This study was made with this setup given its availability in our laboratory.

Three 2D images per cube were acquired, averaged, and flat-field/dark-field corrected. A ROI of 500 × 500 pixels^2^ (1.3 × 1.3 cm^2^) centered on the image of each cube was selected to measure the mean pixel value and its standard deviation. The *μ*_*eff*_ values and their uncertainties (via error propagation) for each cube were computed from the measurements on the images and the measured thickness, using Equation 1. Supplementary Figure S1 contains the *μ*_*eff*_ results for both materials. A weighted chi-square test (95% confidence level) was applied to the *μ*_*eff*_ values of each group of five cubes to check if there exist no statistically significant differences. Because the micro-focus setup and the mammography systems differ in spectrum, irradiation geometry, detector response, and scatter conditions, *μ*_*eff*_ values from both systems are not comparable.

### 3.3 Beam hardening and scatter characterization

Beam hardening and scatter influence the *μ*_*eff*_ values of materials that were experimentally determined as described in Section 3.1. Several empirical models (Kleinschmidt, 1999; Pease et al., 2012) have been proposed in the literature to characterize beam hardening and scatter effects on *μ*_*eff*_ as a function of thickness. Preliminary tests with those models provided the most satisfactory results for the two-parameters model defined as:

$$\mu_{eff}\left(t\right)=\frac{\mu_{0}}{1+kt}$$ (2)

where *μ*_*0*_ represents the effective attenuation coefficient for a very thin slice of material (t→0), and *k* ^*−1*^, the characteristic thickness at which *μ*_*eff*_ is equal to *μ*_*0*_/2. *μ*_*0*_ and *k* are independent parameters estimated simultaneously (weighted nonlinear least squares, based on Equation 2). This phenomenological expression captures the expected monotonic decrease of *μ*_*eff*_ with *t* caused by beam hardening and residual scatter.

## 4 Results

### 4.1 Experimental results

**FIGURE 2:**
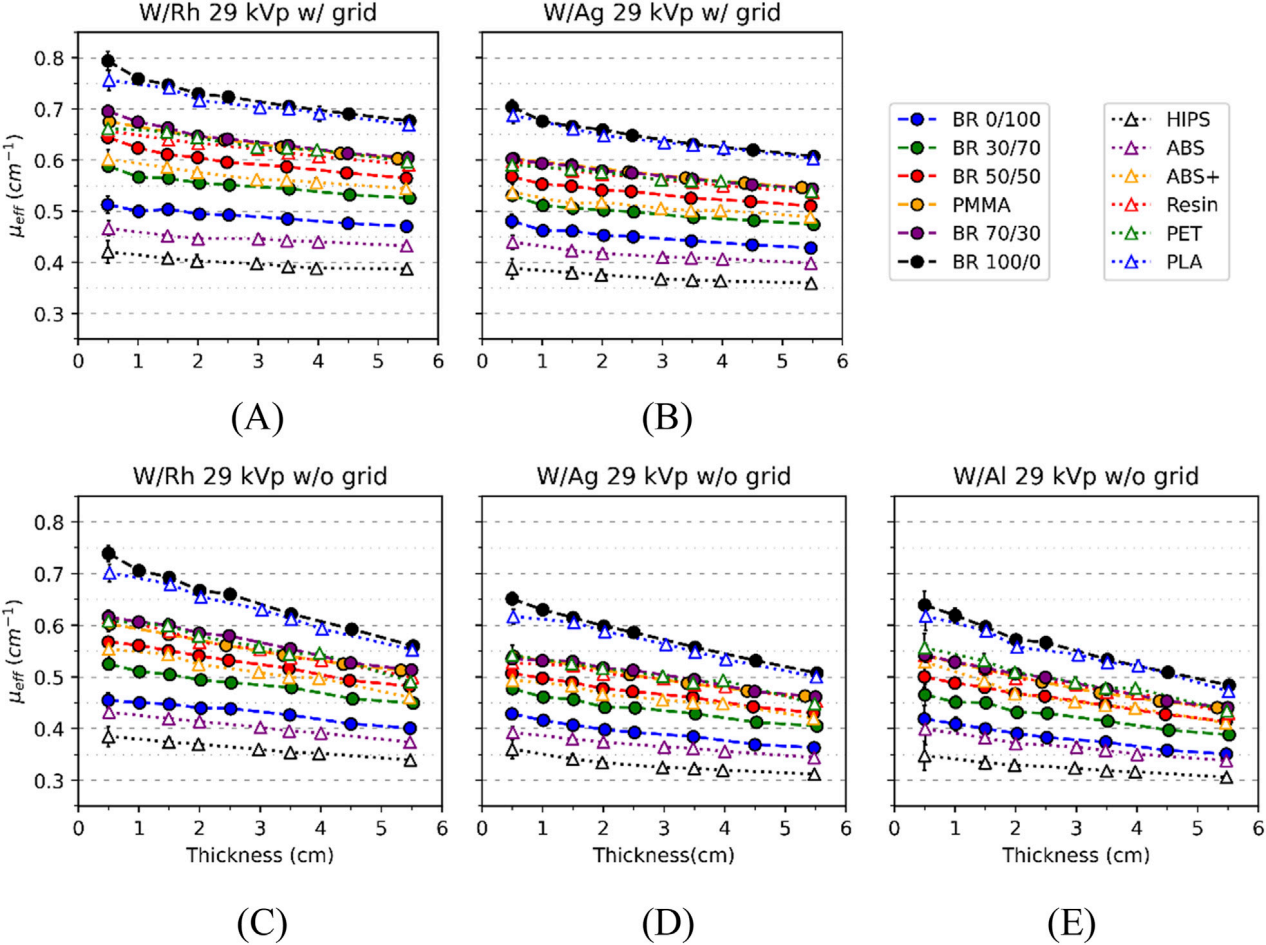
*μ*_*eff*_ versus thickness of reference (filled circle symbols; dashed lines) and 3D materials (open triangle symbols; dotted lines) for M1 system at 29 kVp for DM **(A)** Rh filtration with (w/) and **(C)** without (w/o) grid **(B)** Ag filtration w/and **(D)** w/o grid, and for DBT **(E)** Al filtration w/o grid. Most error bars are not visible (Lines are only for guidance). Error bars show ±1 standard deviation (1σ).

For all acquisition conditions, the effective X-ray attenuation coefficients (*μ*_*eff*_) were determined for both reference and 3D materials across the full range of thicknesses, as described in Section 3.1. Figure 2 shows *μ*_*eff*_ values obtained with the M1 system at 29 kVp for DM (Rh and Ag filtrations; w/and w/o grid) and DBT (Al filtration; w/o grid). Results for 27 and 31 kVp are shown in Supplementary Figure S2 and Supplementary Figure S3.

The relative uncertainties of *μ*_*eff*_ were approximately 1% for all thicknesses, except for the 0.5 cm step, where they increased to about 4%. This higher uncertainty is mainly due to the smaller differences between the pixel values measured for the 0.5 cm thickness and those corresponding to the flat-field image (0 cm), combined with the larger relative error in the measurement of the 0.5 cm step thickness.

Figure 2 exhibits that *μ*_*eff*_ values decrease monotonically with thickness for all materials and acquisition conditions. The reference materials simulating higher glandular composition consistently show greater *μ*_*eff*_ values across all the experimental conditions. The largest reductions of *μ*_*eff*_ with increasing thickness are for more attenuating materials such as BR 100/0 and PLA, while the smallest variations are for the less attenuating materials such as BR 0/100, ABS or HIPS. For all materials, from 1.0 to 5.5 cm, *μ*_*eff*_ values decrease within 6%–14% and 12%–28% ranges for w/grid and w/o grid cases, respectively. The removal of the anti-scatter grid (w/o grid cases) further reduces the *μ*_*eff*_ within a range of 47%–76%.

**FIGURE 3:**
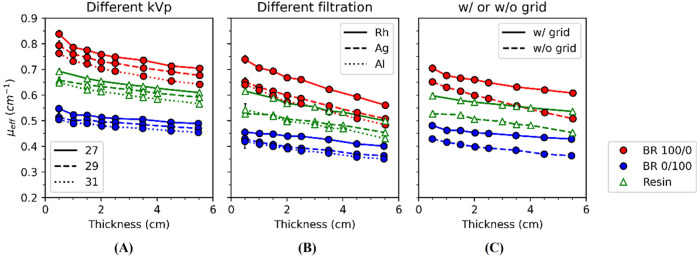
Dependence of *μ_eff_* values for **(A)** different kVp (keeping W/Rh w/grid), **(B)** filtration (keeping 29 kVp w/o grid) and **(C)** with (w/) and without (w/o) anti-scatter grid (keeping W/Rh 29 kVp) for BR 100/0, BR 0/100 and resin (Lines are only for guidance). Error bars show ±1 standard deviation (1σ).

For BR 100/0, BR 0/100 and resin, the independent effects on *μ*_*eff*_ values due to the selection of kVp, filtration and the use of the anti-scatter grid are shown in Figure 3. As expected, *μ*_*eff*_ curves decrease as kVp increases (Figure 3A) and when the anti-scatter grid is in place (Figure 3C). The *μ_eff_* values are lowest for Al, intermediate for Ag, and highest for Rh filters, although the curves corresponding to Al and Ag are visually similar (Figure 3B). On the one hand, Figure 2 allows the visualization of 3D-reference material pairings with similar *μ*_*eff*_ curves. Specifically, the following pairings were identified: (BR 100/0 - PLA), (BR 70/30 - resin, PET) and (BR 30/70, BR 50/50 - ABS+). The PMMA - BR 70/30 pairing of both reference materials was also identified. On the other hand, ABS showed deviations of up to 12% relative to BR 0/100, while HIPS exhibited the largest overall mismatch across conditions (Figure 2).

**FIGURE 4:**
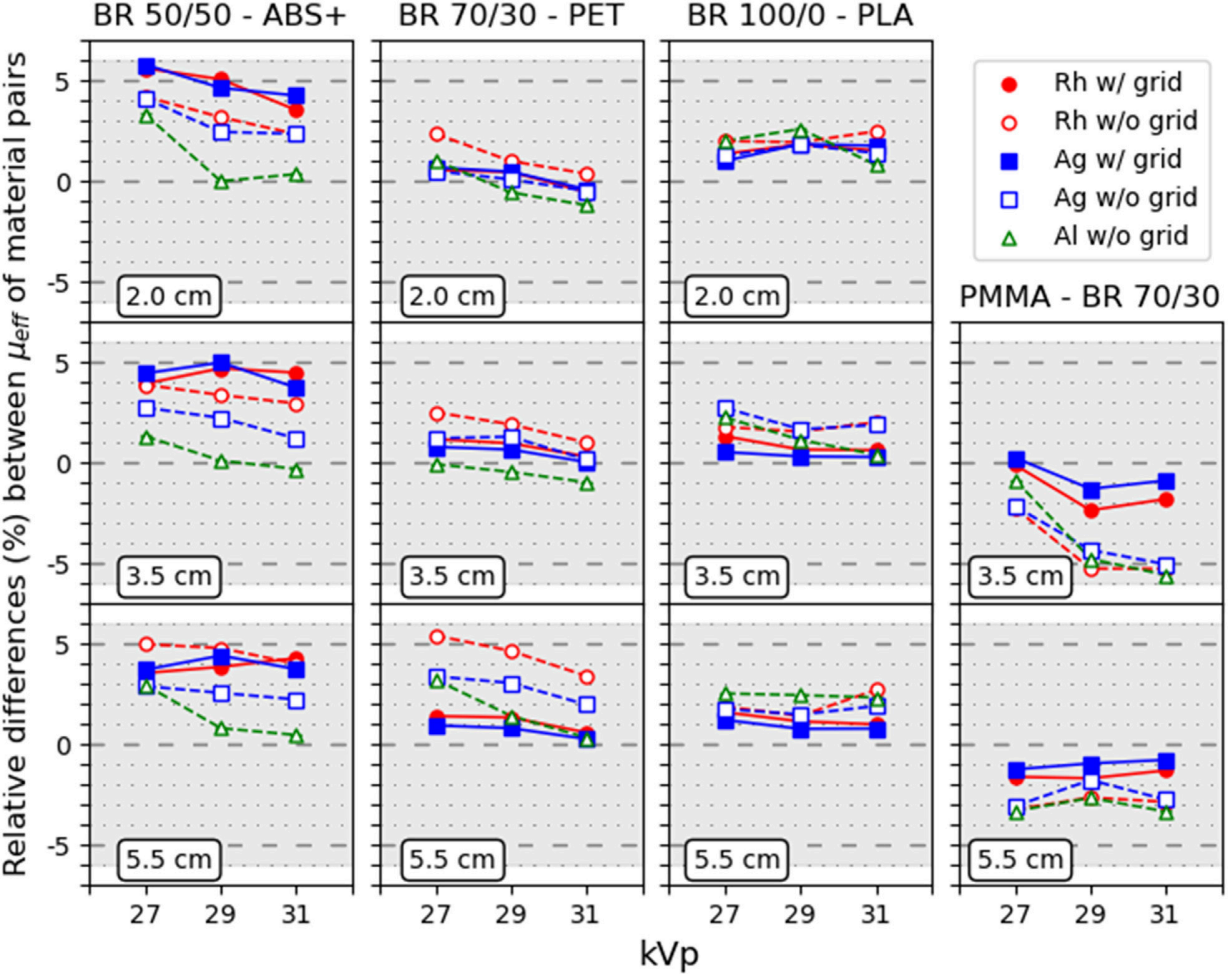
Relative differences (%) between *μ*_*eff*_ of the selected material versus the kVp values, for 2.0, 3.5 and 5.5 cm step thicknesses for M1. The shaded region correspond to a ±6% range. Error bars show ±1 standard deviation (1σ).

Quantitative analysis at small (2.0 cm), medium (3.5 cm), and large (5.5 cm) thicknesses revealed that reference–3D material pairings maintained relative differences in *μ*_*eff*_ within ±6% across all acquisition conditions (Figure 4). Additionally, PMMA consistently aligned closely with BR 70/30, with relative differences ranging from −4% to 1% under all acquisition conditions considered (Figure 4).

**FIGURE 5:**
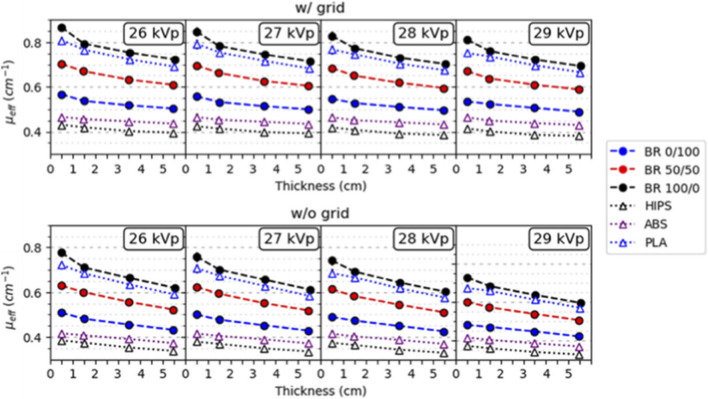
*μ*_*eff*_ versus thickness of reference (filled symbols; dashed lines) and 3D (open symbols; dotted lines) materials obtained with M2 system at 26–29 kVp range with (w/) and without (w/o) grid. Error bars are not visible (Lines are only for guidance).

For the M2 system, Figure 5 shows the *μ*_*eff*_ values of reference and 3D materials for W/Rh 26–29 kVp, w/and w/o grid. (Note: In M2, DM and DBT employs same filtration). The relative uncertainties (included in the plots) are around 1% for all thicknesses, except for 0.5 cm (3%), similar to the ones found for M1. As expected, *μ*_*eff*_ values for M2 show the same trend with material thickness, kVp and presence/absence of the grid as observed for M1 (Figures 2, 3).

Effective attenuation coefficients at 0.5, 1.5, 3.5, and 5.5 cm were compared between M1 and M2 under identical acquisition conditions (W/Rh at 27 and 29 kVp w/grid), for reference (BR 0/100, BR 50/50, BR 100/0) and 3D (HIPS, ABS, and PLA) materials. A weighted chi-square test for paired data showed no statistically significant differences at the 95% confidence level. The results of the weighted chi-square test for paired data demonstrate that there is no statistically significant differences (p > 0.05). Therefore, the equivalence observed between 3D and reference materials in M1 can be extended to M2.

### 4.2 Beam hardening and scatter characterization

The (*μ*_*0*_, *k*) parameters were derived from the fittings of *μ*_*eff*_ values to the empirical model defined by Equation 2, obtaining *R* ^*2*^ values >0.99. The fit parameters for all materials are listed in Supplementary Table S1 and Supplementary Table S2. Furthermore, the resulting fittings curves for all target/filter combinations at 29 kVp are shown in Supplementary Figure S4; similar trends were observed at 27 and 31 kVp. Considering all cases, the average relative uncertainties are 1% and 20% for *μ*_*0*_ and *k*, respectively. To estimate the precision of the model, for all acquisition conditions, materials, and thicknesses considered, the relative uncertainties of the *μ*_*eff*_ values obtained with the model were calculated—considering the uncertainties of *μ*_*0*_ and *k*—and compared with those of the experimental *μ*_*eff*_ values. For the fitting model, the relative uncertainties were 2.3% on average (range: 0.5%–11.3%), whereas for the experimental measurements they were 1.4% on average (range: 0.4%–8.2%). Therefore, despite the higher uncertainty associated with *k*, the predictions of the model and the experimental measurements exhibit a similar level of precision.

**FIGURE 6:**
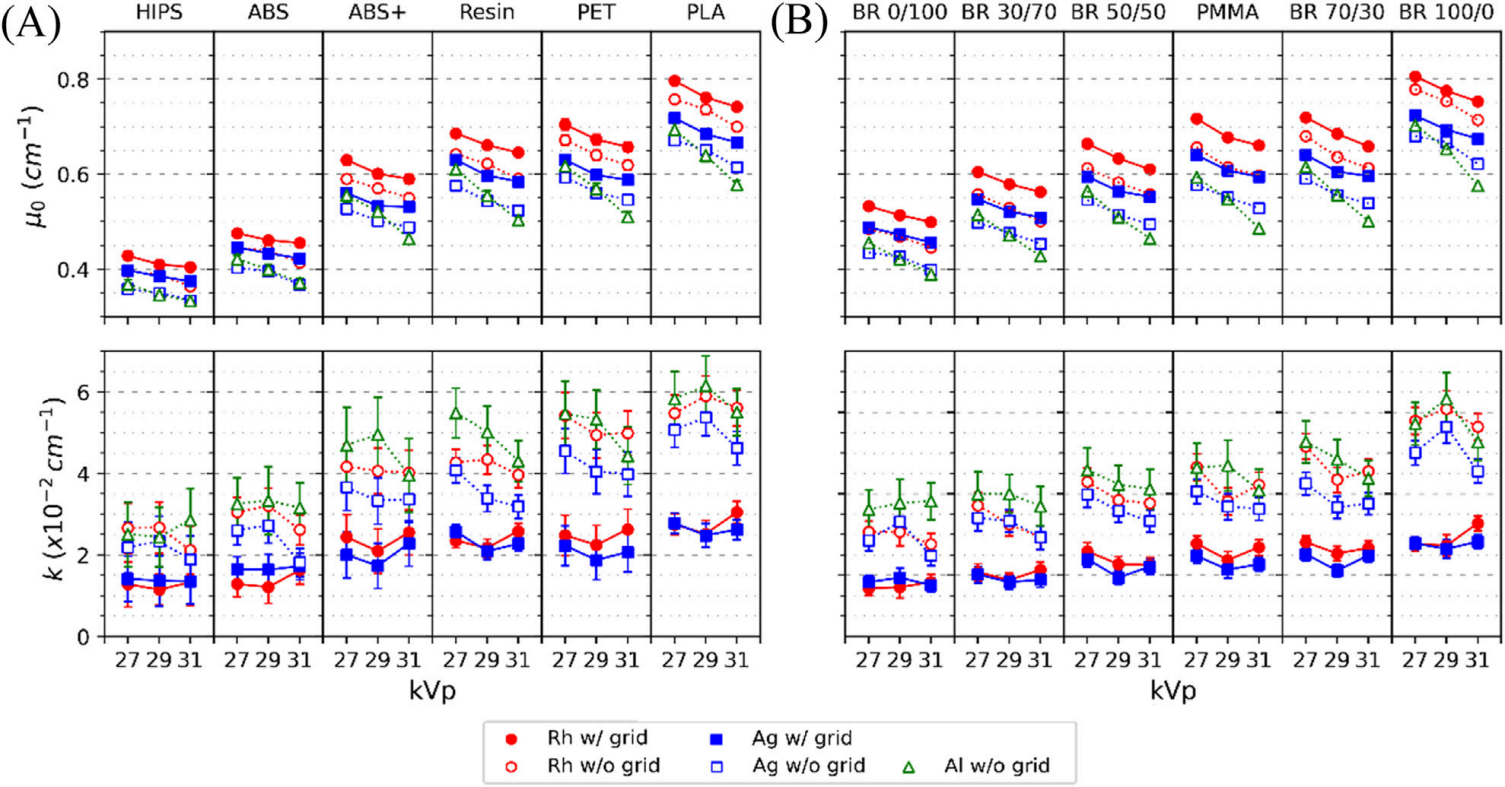
*μ*_*0*_ and *k* values versus kVp for **(A)** 3D and **(B)** reference materials obtained for M1 (Lines are only for guidance). Error bars show ±1 standard deviation (1σ).

In addition, Figure 6 shows the (*μ*_*0*_, *k*) values plotted against kVp for all the materials, spectra and w/and w/o grid cases. As expected, *μ*_*0*_ decreased with increasing kVp, and is consistently higher for w/grid acquisitions, particularly with Rh filtration. The *μ*_*eff*_ ranking among materials observed in Section 4.1 was preserved for *μ*_*0*_ values. The *k* parameter is approximately twice as high w/o the grid. As shown in Figure 6, *k* increased with material attenuation, especially in the w/o grid cases. The influence of beam spectra on *k* values can be considered negligible when accounting for the uncertainties associated with *k*.

**FIGURE 7:**
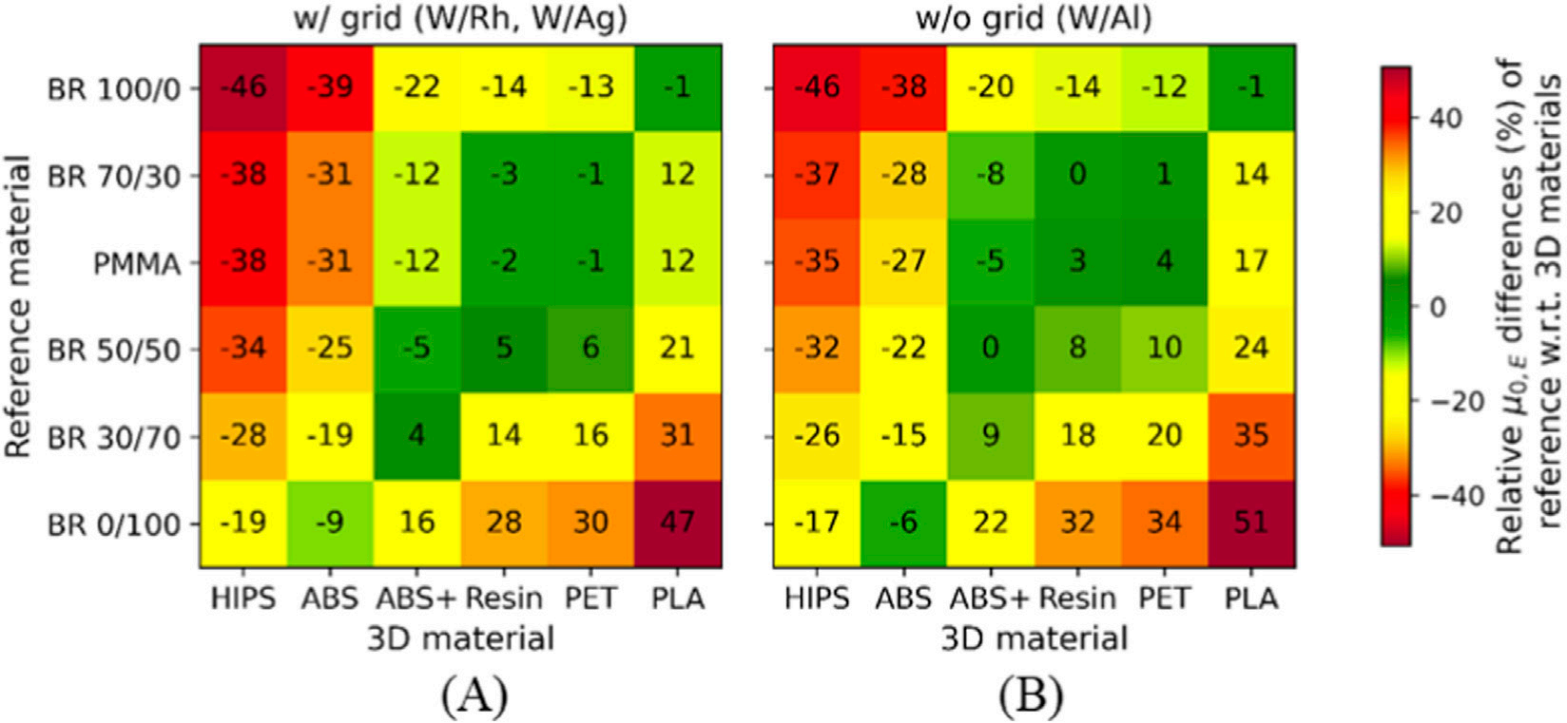
Heat map with cell values corresponding to the *µ*_*0*_ relative differences between reference and 3D materials averaged over all the exposure conditions **(A)** with (w/) and **(B)** without (w/o) anti-scatter grid for M1 system.

For M1, relative differences of *μ*_*0*_ values between reference and 3D materials were calculated under all acquisition conditions (W/Rh, W/Ag, and W/Al at 27, 29, and 31 kVp, w/and w/o grid). These values were then averaged separately for the grid conditions (W/Rh and W/Ag w/grid) and for the no-grid conditions (W/Rh, W/Ag, and W/Al w/o grid). These values are shown as heat maps in Figures 7A,B. The results confirm that the potential reference–3D material pairings with equivalent X-ray attenuation (±6%) remain valid, even when the grid is removed.

**FIGURE 8:**
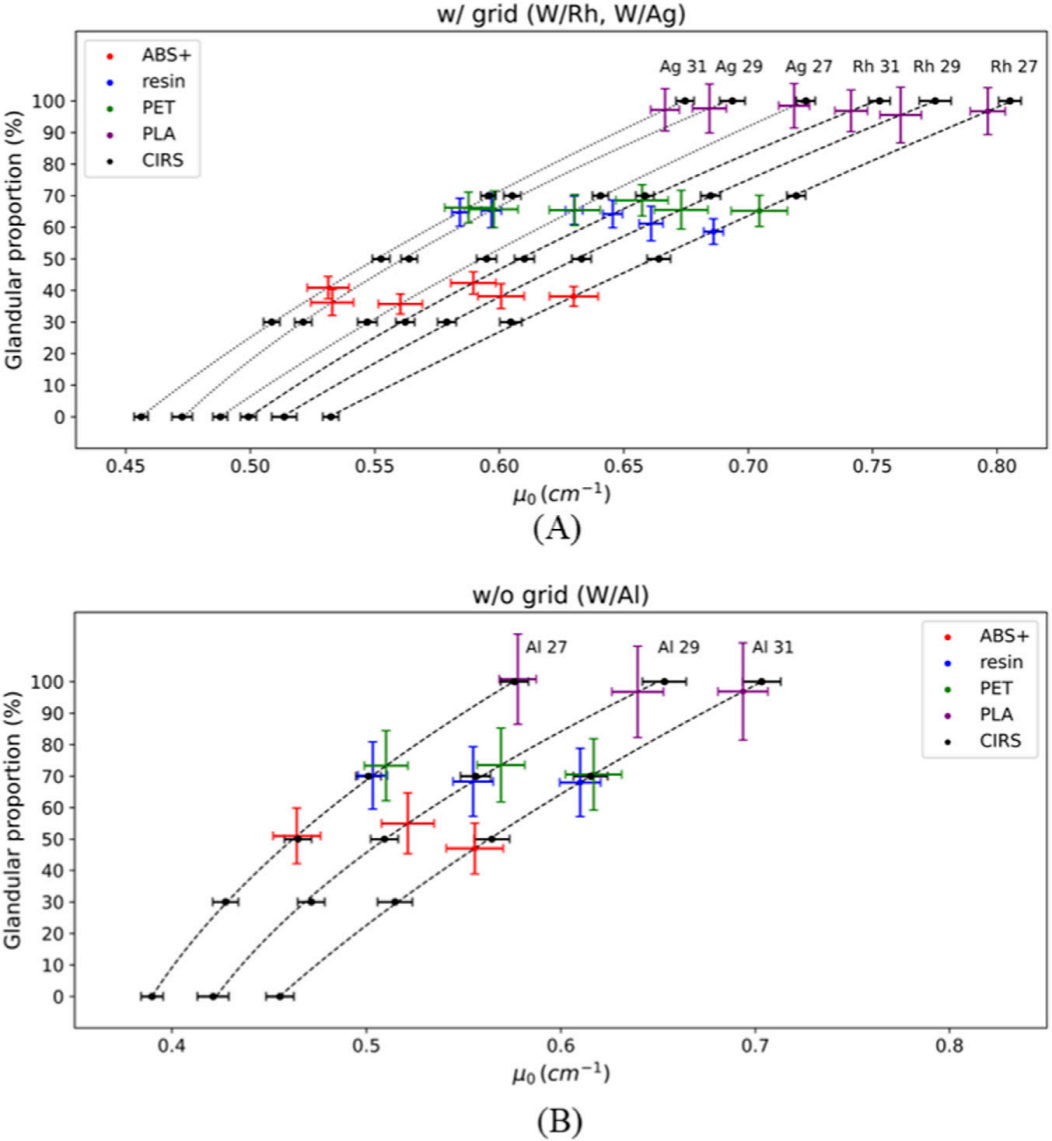
Equivalent glandular proportions of the 3D materials for **(A)** w/and **(B)** w/o grid cases. Lines correspond to fittings between *μ*_*0*_ values and known breast glandular/adipose ratio (BR)of CIRS materials for each filter and kVp combination (dashed lines for rhodium (Rh) and aluminum (Al) filtration, dotted lines for silver (Ag) filtration). Horizontal error bars correspond to the uncertainties of *μ*_*0*_ of the empirical model (Equation 2). Error bars show ±1 standard deviation (1σ).

*μ_0_* values obtained for the CIRS materials were fitted to their known glandular proportion (0%–100%) using a quadratic function (weighted least squares) for each acquisition condition, yielding R^2^ > 0.9993 for all the fits. These resulting fittings are shown in Figure 8A (w/grid) and Figure 8B (w/o grid). For each acquisition condition, the corresponding glandular proportion of the 3D materials (ABS+, resin, PET and PLA) were derived by substituting their *μ*_*0*_ values into their respective fitting equations. These fittings thus enable an effective determination of the range of glandular tissue proportion to which a 3D-printed material is equivalent, based on its corresponding *μ*_*0*_ value. The equivalent glandular proportions and uncertainties of the 3D-printed materials are included in Table 2.

**TABLE 2:** Equivalent glandular proportions (%) corresponding to the 3D-printed materials obtained from the fittings between *μ*_*0*_ values and known breast glandular/adipose ratio of CIRS materials.

3D-printed material	Equivalent glandular proportion (%)								
	W/Rh w/grid			W/Ag w/grid			W/Al w/o grid		
	27 kVp	29 kVp	31 kVp	27 kVp	29 kVp	31 kVp	27 kVp	29 kVp	31 kVp
ABS+	38 ± 3	38 ± 4	42 ± 4	36 ± 3	36 ± 4	41 ± 4	47 ± 8	55 ± 10	51 ± 9
Resin	59 ± 4	61 ± 5	64 ± 4	65 ± 5	65 ± 5	65 ± 4	68 ± 11	68 ± 11	70 ± 11
PET	65 ± 5	66 ± 6	69 ± 5	65 ± 5	66 ± 6	66 ± 5	71 ± 11	64 ± 12	73 ± 11
PLA	97 ± 7	96 ± 9	97 ± 7	98 ± 7	98 ± 7	97 ± 7	97 ± 15	97 ± 15	101 ± 14

W, tungsten; Rh, rhodium; Ag, silver; Al, aluminum; w/grid, with the anti-scatter grid; w/o grid, without the anti-scatter grid. The uncertainties correspond to 1-σ.

From the attenuation curves (Figure 2), HIPS and ABS (for the specific color and manufacturer evaluated) showed that the X-ray attenuation values are lower than those of CIRS BR 0/100 (equivalent to adipose tissue). As they lie outside the attenuation range used for the polynomial fits (BR 0/100–BR 100/0), no meaningful equivalent glandular proportion could be assigned, and these materials were therefore excluded from the equivalence analysis presented in Figure 8 and Table 2. By considering the uncertainties, equivalent glandular proportions of all the materials are kept constant for W/Rh and W/Ag spectra. However, the uncertainties of equivalent glandular proportions are higher for all the W/Al spectra due to the higher uncertainties in the corresponding *μ*_*0*_ values, because of the greater contribution of scatter as the anti-scatter grid is not in place during the acquisition.

## 5 Discussion

Previous studies have reported the X-ray attenuation properties of 3D-printed materials using CT setups (Dancewicz et al., 2017; Ma et al., 2021; Kozee et al., 2023), synchrotron radiation (Ivanov et al., 2018; Mettivier et al., 2022), or standardized diagnostic X-ray qualities (International Electrotechnical Commission (IEC), 2025; Kunert et al., 2022; Savi et al., 2021; Villani et al., 2021). More recent investigations have employed beam qualities closer to mammography and, in some cases, even mammography systems (Oliveira et al., 2022), but often required additional instrumentation beyond the system itself. Furthermore, they often lacked simultaneous benchmarking against reference materials specifically designed to mimic breast glandular and adipose tissues. In this work, attenuation was measured directly in two commercial mammography and tomosynthesis systems (Hologic 3Dimensions and Siemens Mammomat Inspiration), relying only on the installed X-ray tube and detector, without any external devices. This makes the methodology simple, accessible, and reproducible in hospital or clinical environments where 3D-printed phantoms may be designed and fabricated. Importantly, both candidate 3D-printing materials and breast tissue–equivalent references (CIRS and PMMA plates) were imaged under identical conditions in the same systems, enabling robust benchmarking. Note that *μ*_*eff*_ values obtained in this work (Equation 1) are affected by beam hardening and scatter; thus, we used them to compare attenuation properties between 3D-printed and reference materials under same acquisition conditions. For materials with similar *μ*_*eff*_ *(t)*, the scattered radiation acts as a common bias. Furthermore, *μ*_*eff*_ *(t)* values were fitted to an empirical model (Equation 2) which allowed the characterization of beam hardening and scatter effects in a practical manner. The *μ*_*eff*_ values of reference materials in the Hologic and Siemens systems may also serve as baseline standards for future evaluations of additional 3D-printing materials, if measurements are performed on systems that share similar technical characteristics.

As expected, the effective attenuation coefficients (*μ*_*eff*_) measured for all materials decreased with increasing thickness, reflecting the combined effects of beam hardening and scatter (Figures 2, 3, 5). The magnitude of this reduction depends on material composition and acquisition conditions. Consistently, highly attenuating materials such as PLA and BR 100/0 exhibited the strongest decreases of *μ*_*eff*_ with thickness, while ABS, HIPS, and BR 0/100 showed a softer decrease. Removal of the anti-scatter grid had the most pronounced effect, reducing *μ*_*eff*_ by up to a factor of around two for all cases (Figure 3).

Consistent pairings between 3D and reference materials were identified, as seen in Figures 4, 7. PLA was equivalent to highly glandular tissue (BR 100/0), resin and PET matched intermediate compositions (BR 70/30), and ABS+ aligned with adipose-dominant mixtures (BR 30/70–50/50). As expected, PMMA closely matched BR 70/30 across all conditions, confirming its long-established role as a surrogate for average breast tissue. Conversely, HIPS and uncolored ABS showed larger mismatches, suggesting that not all widely available filaments are suitable tissue substitutes. The results demonstrate that common, low-cost materials such as PLA, PET, resin, and ABS+ can reproduce the attenuation behavior of different breast tissue compositions, enabling their use for anthropomorphic phantom fabrication (Ivanov et al., 2018; Mettivier et al., 2022).

**FIGURE 9:**
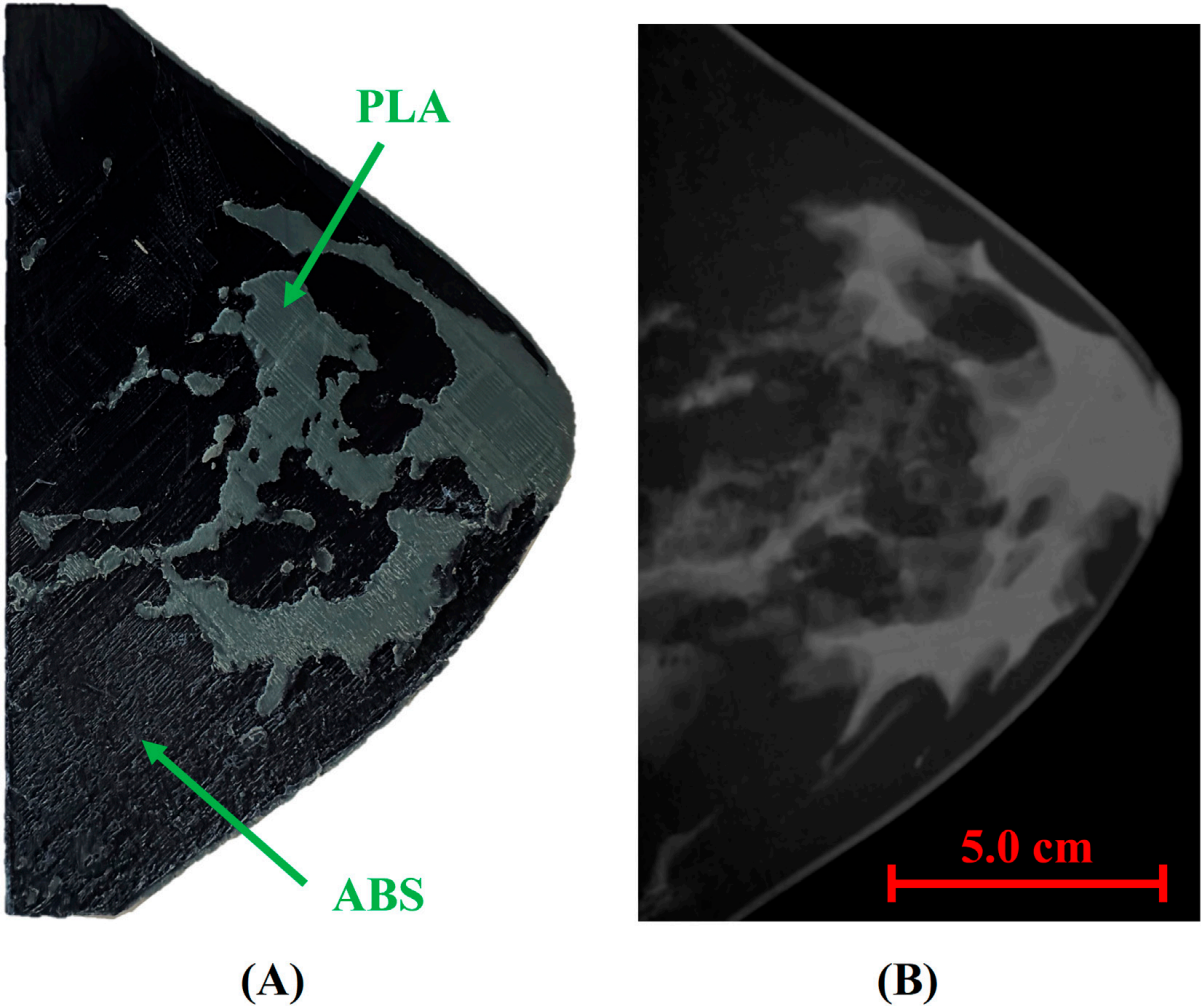
**(A)** 3D-printed 11-mm-thick slab printed with PLA for gland + skin and ABS for fat, following the attenuation equivalences established in this study **(B)** and a digital mammography of the phantom slab digital mammography image acquired on a Hologic 3Dimensions.

As a step toward anthropomorphic validation, we include in Figure 9A an 11-mm slice 3D-printed with PLA (glandular + skin) and ABS (adipose tissue) (Belarra et al., 2025), being the selection of materials according to the *μ*_*eff*_ equivalences reported in this work. Also, a digital mammography of the slab is shown in Figure 9B. Beyond this example, our group has produced a complete multi-slice anthropomorphic phantom and acquired DM, DBT, and micro-CT images; a comprehensive evaluation of anatomical realism, print quality, and task-based performance will be presented in follow-up work.

Another important finding is the robustness of material equivalence across two different vendor systems (Figures 2, 5). The absence of statistically significant differences between M1 and M2 confirms that the proposed characterization method is system-independent, however, both systems share same anode (W), filter (Rh 0.05 mm) and detector technology (a-Se).

The empirical two-parameter model (Kleinschmidt, 1999; Pease et al., 2012) used here (*μ*_*0*_, *k*) provided an excellent fit to the experimental data (*R* ^*2*^ *> 0.99*), offering a practical framework to describe beam hardening and scatter effects. The *μ*_*0*_ parameter preserved the attenuation ranking across materials and acquisition conditions (Figure 6) and proved to be a reliable predictor of equivalent glandular proportion when mapped against CIRS calibration curves. Through this approach, equivalent glandular proportions were assigned to 3D-printed materials (Figure 8), providing a quantitative pathway to select printing materials according to the breast tissue composition to be mimicked. Although *k* values carried larger uncertainties, the overall predictive precision of the model to obtain *μ*_*eff*_ values for a certain thickness was comparable to that of direct experimental measurements. This demonstrate that this two-parameters model is suitable for this application.

Future work will extend this approach to a broader range of 3D printing materials and mammography systems with diverse beam qualities, aiming to confirm the consistency of equivalences across platforms.

## 6 Conclusion

The proposed framework for the characterization of attenuation properties of 3D-printed materials provides robust and clinically relevant estimates of the effective X-ray attenuation coefficient (*μ*_*eff*_). This validates the use of routine digital mammography and tomosynthesis systems, without additional instrumentation, for reliable material characterization.

Our results demonstrate that several 3D-printed polymers exhibit attenuation properties comparable to breast tissue-equivalent reference materials across multiple kVp settings, filtration types, and two clinical mammography systems from different manufacturers. Specifically, grey PLA shows excellent agreement with BR 100/0 (glandular tissue), grey PET and resin align with BR 70/30 or PMMA, and ABS+ presents intermediate attenuation between BR 30/70 and BR 50/50. The derived *μ_0_* parameters can be interpreted as monoenergetic attenuation coefficients in the absence of scatter, and closely match values reported in the literature using spectrometry and synchrotron techniques (Byng et al., 1998; Mettivier et al., 2022).

Overall, this study advances the feasibility of locally producing customized anthropomorphic breast phantoms within hospital environments, thereby supporting realistic image quality evaluation and optimization in clinical practice.

## Data Availability

The original contributions presented in the study are included in the article/Supplementary Material, further inquiries can be directed to the corresponding author.
