# Supplementary material for: Experimental determination of effective X-ray attenuation coefficients of 3D-printed materials under clinical mammography spectra
Source: Front Bioeng Biotechnol. 2026 Feb 5;13:1719551. doi: 10.3389/fbioe.2025.1719551 (PMC12916599; doi:10.3389/fbioe.2025.1719551)
Supplement: Supplementary file 1 [file Supplementaryfile1.docx]

Supplementary Material

# Supplementary Figures and Tables

## Supplementary Figures

| **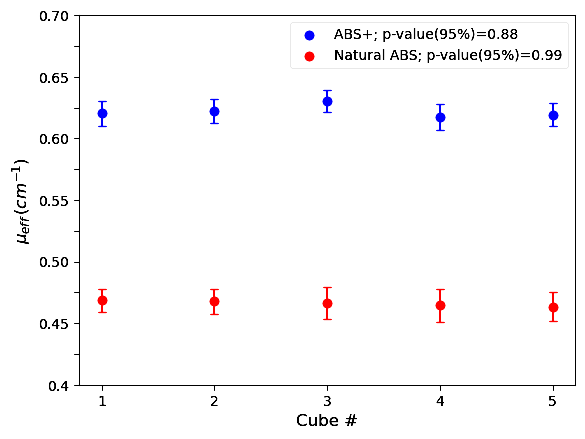** |
| --- |
| **Supplementary Figure 1.** Printing reproducibility of ABS+ with the Bambu X1C (blue symbols) and white ABS with the BCN3D Sigma R19 (red symbols). |

| **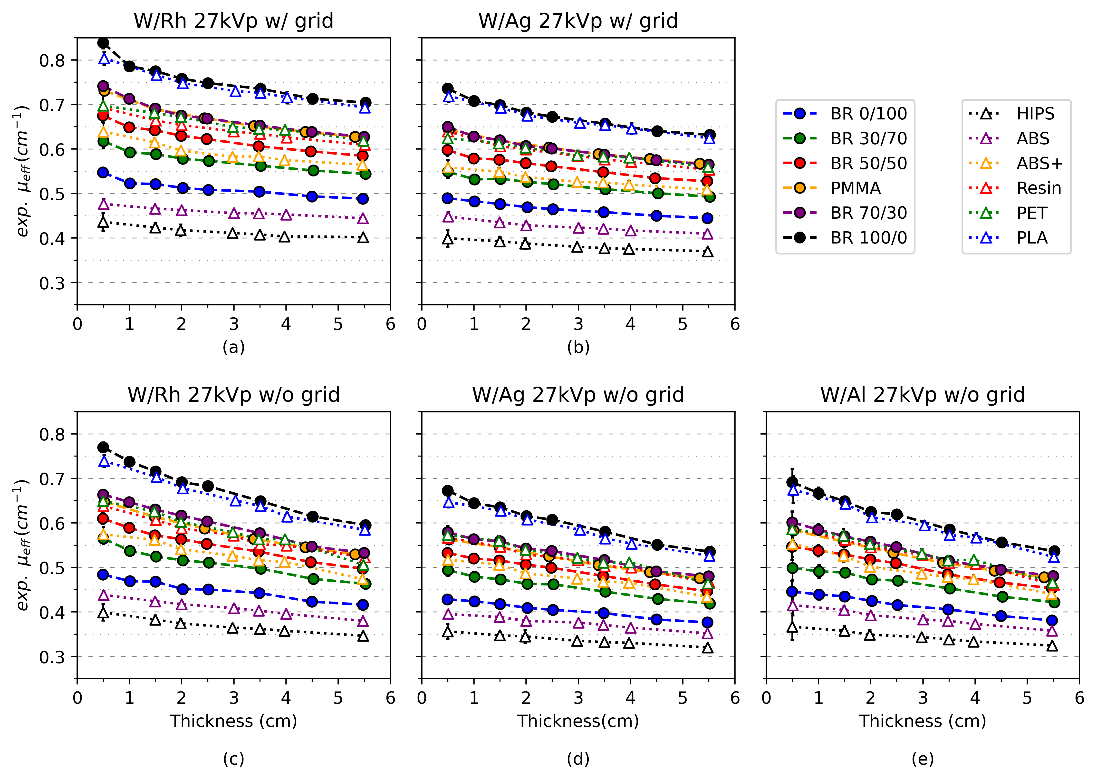** | **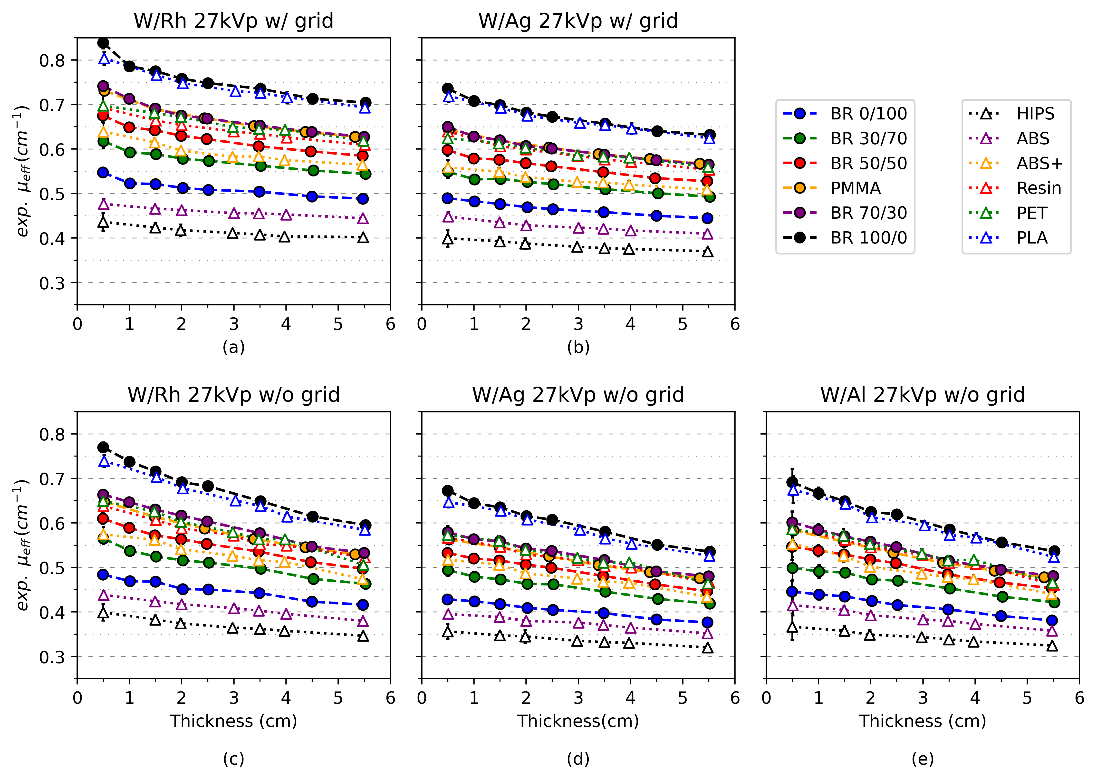** | |
| --- | --- | --- |
| **(A)** | **(B)** |  |
| **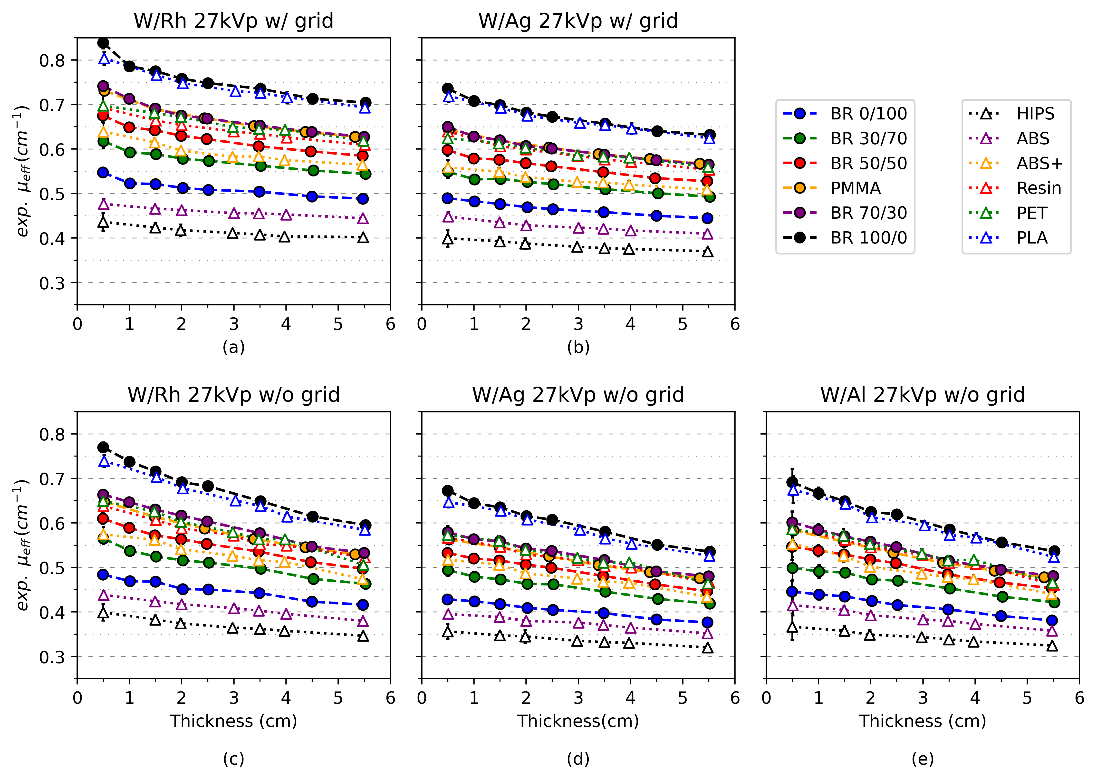** | **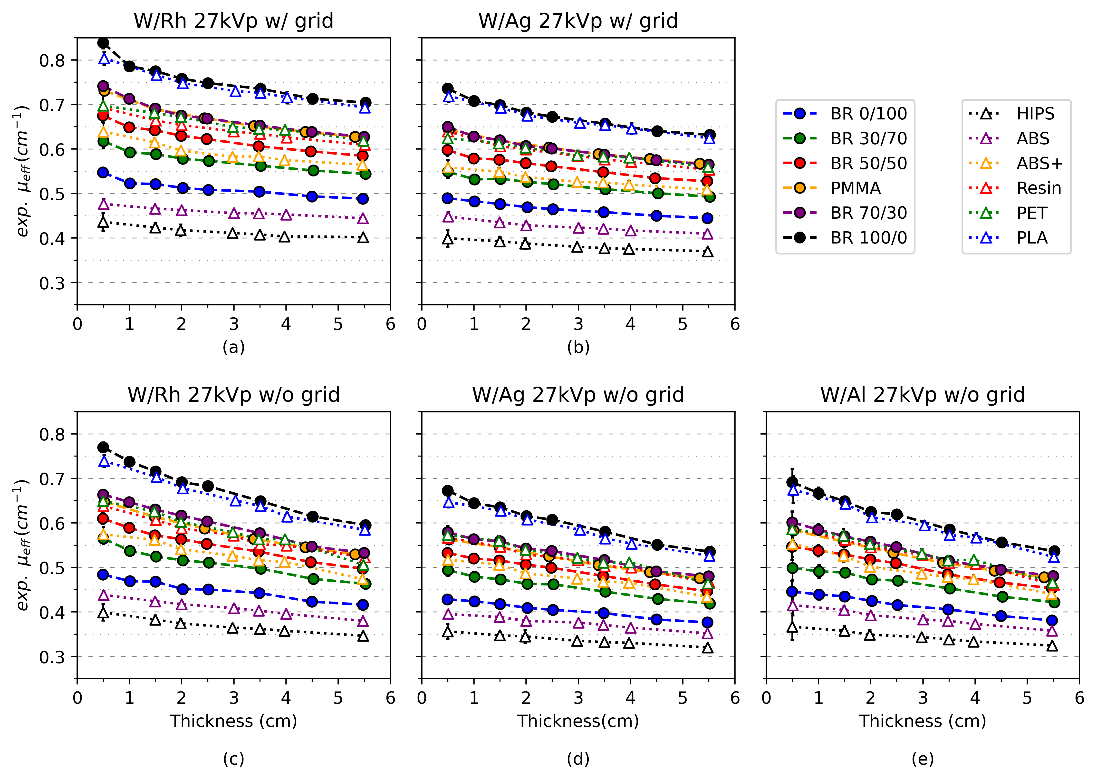** | **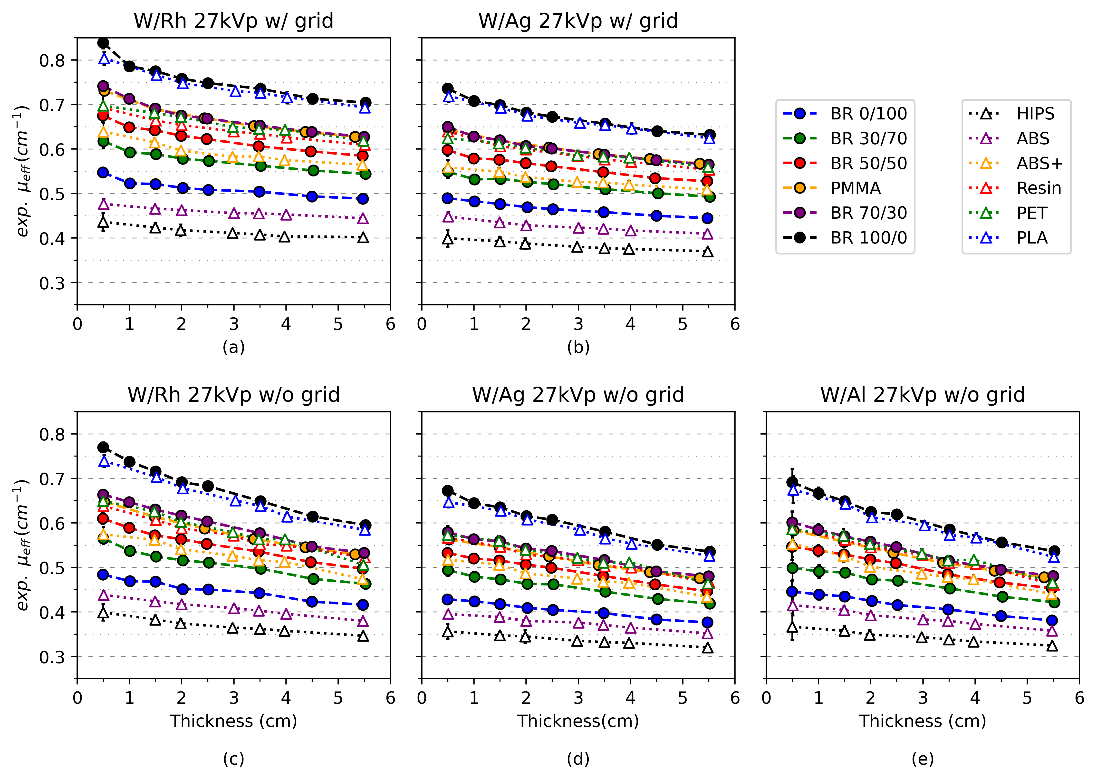** |
| **(C)** | **(D)** | **(E)** |
| **Supplementary Figure 2**. Experimental effective attenuation coefficients (*μ_eff_*) of reference (filled symbols-dashed lines) and 3D printed materials (open symbols-dotted lines) for DM at 27 kVp and: (A) Rh filtration with grid (w/ grid) and (C) without grid (w/o grid); (B) Ag filtration w/ grid and (D) w/o grid, and for DBT at 27 kVp and (E) Al filtration w/o grid. Error bars are not visible except for the thickness of 0.5 cm. (Lines are only for guidance). | | |

| **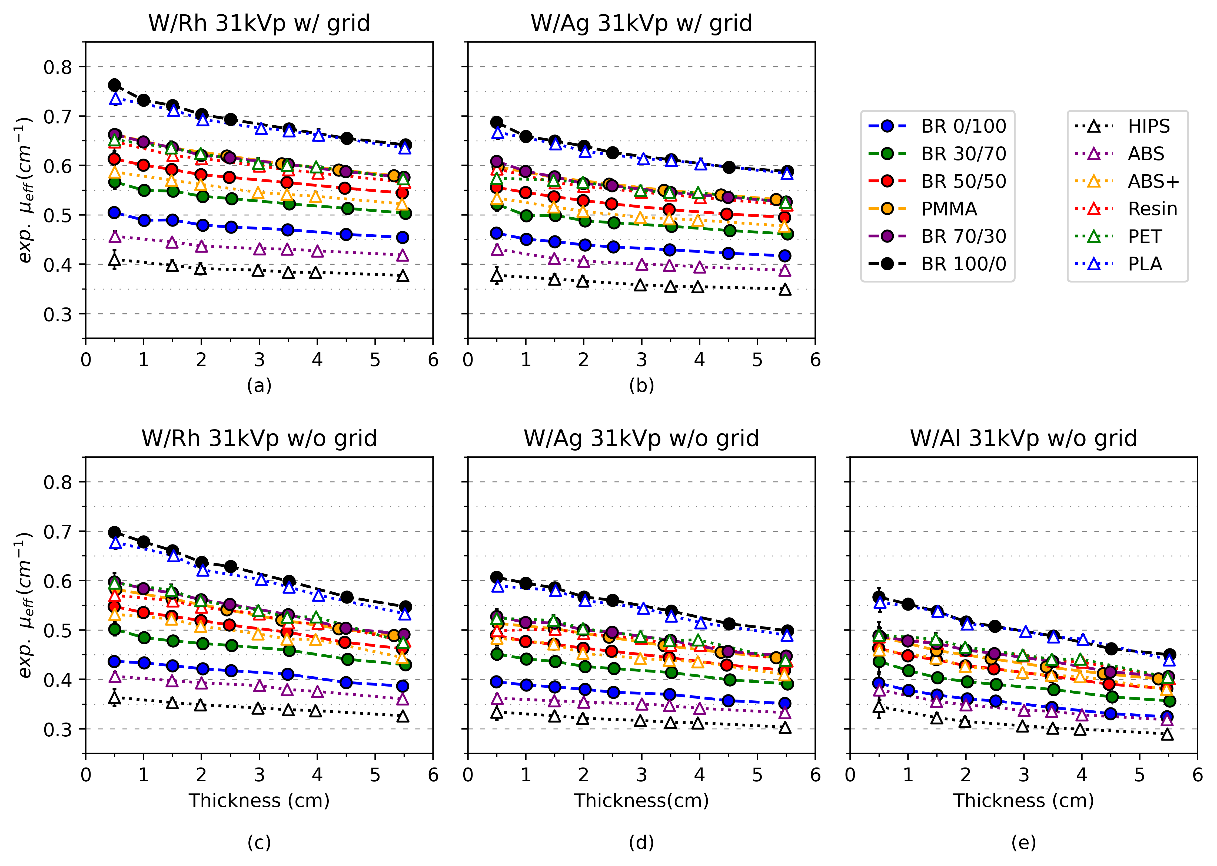** | **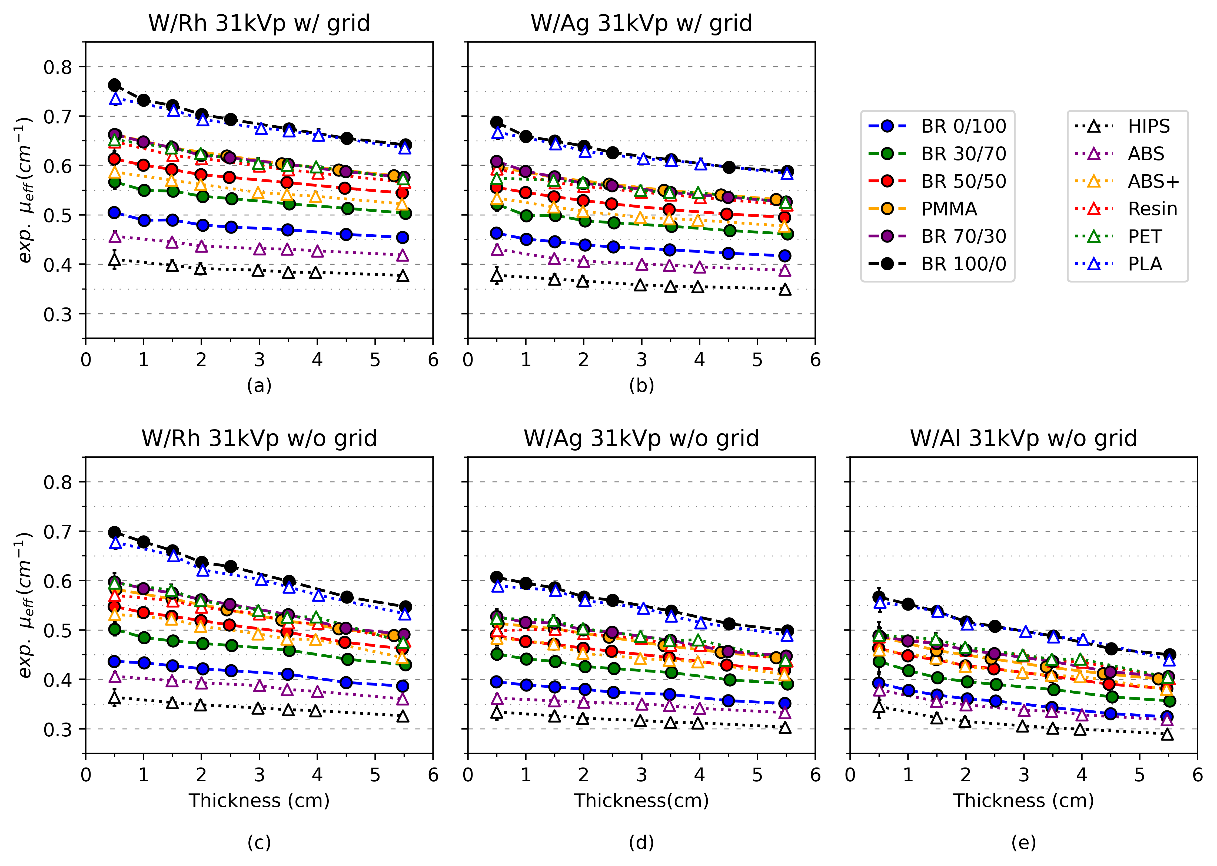** | |
| --- | --- | --- |
| **(A)** | **(B)** |  |
| **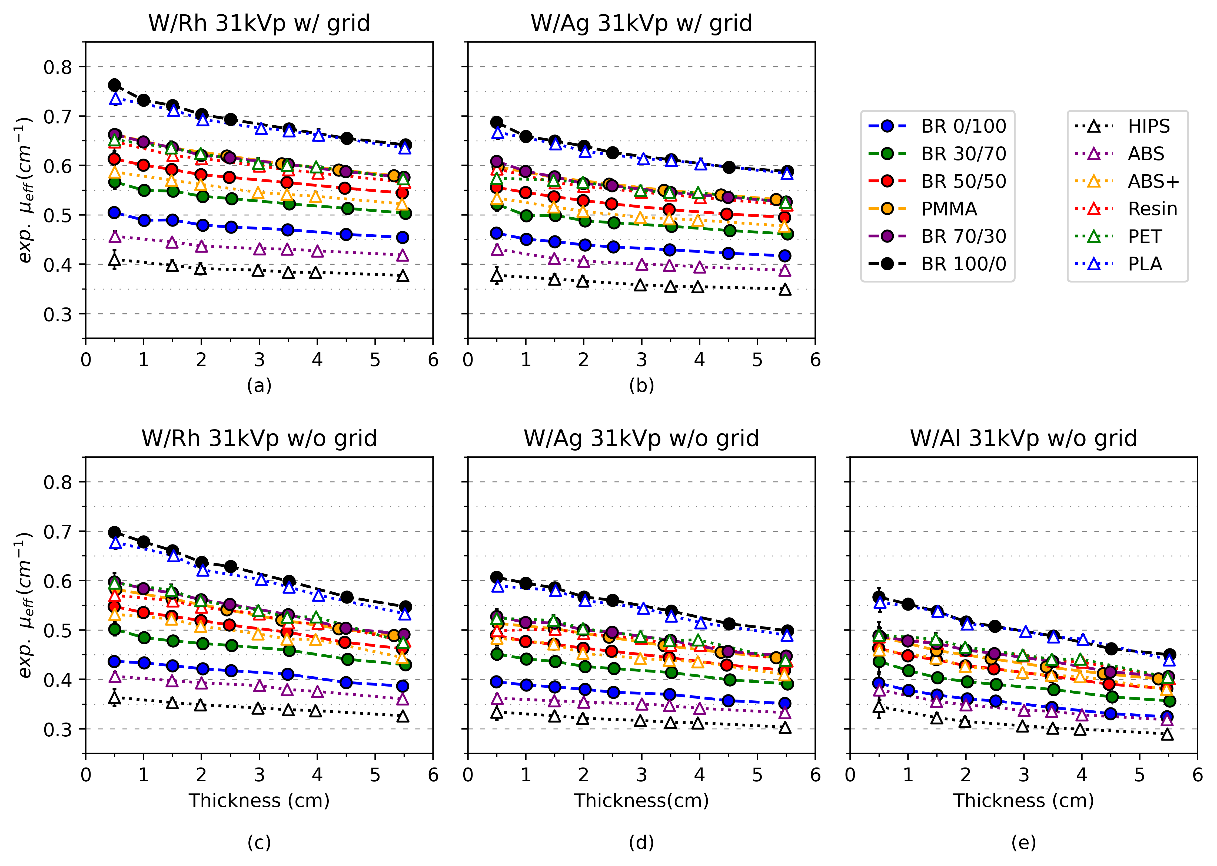** | **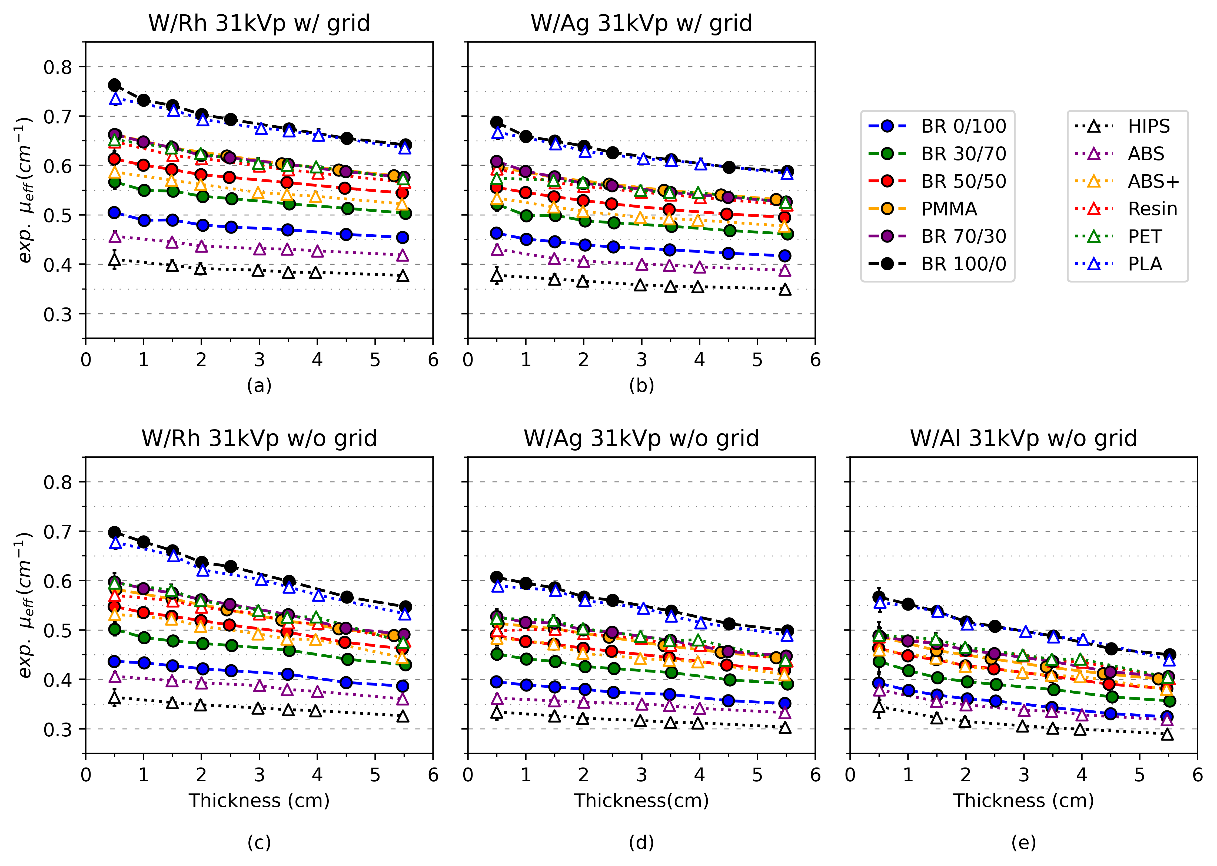** | **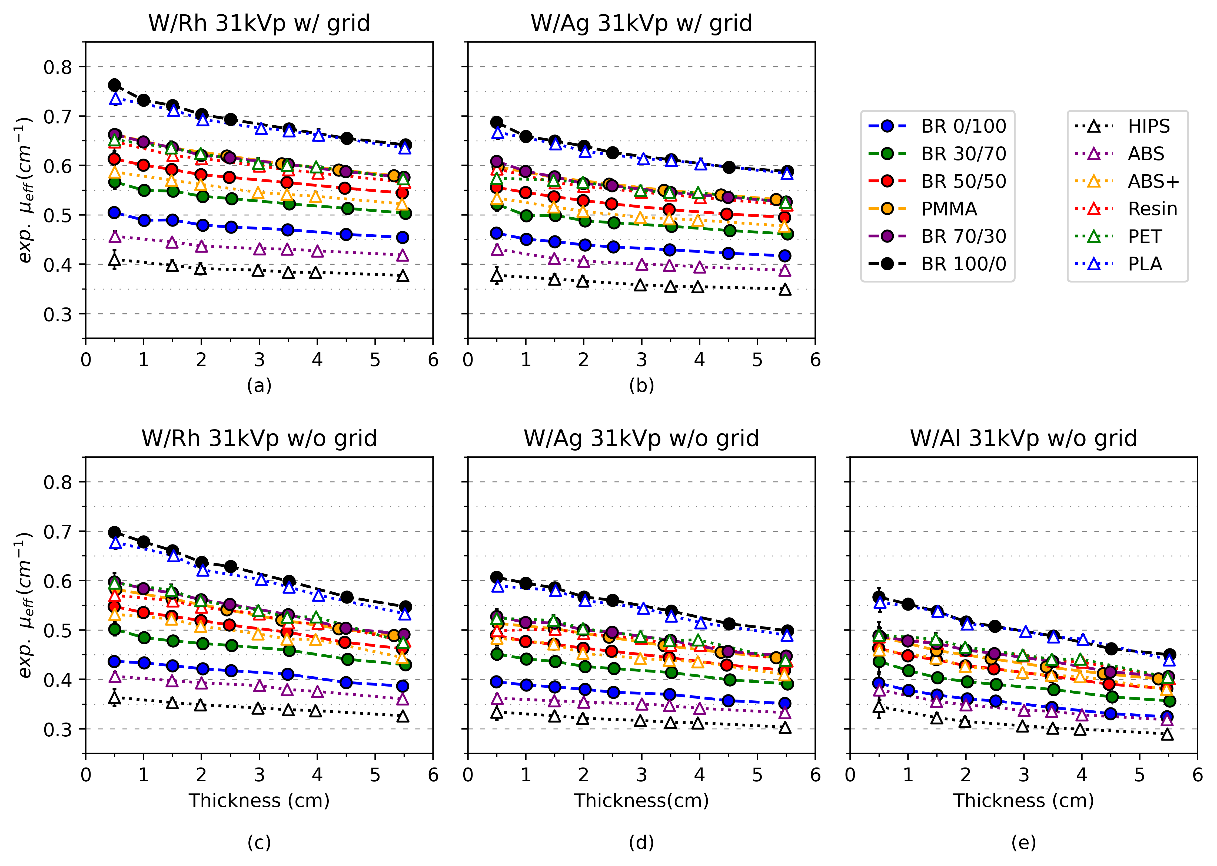** |
| **(C)** | **(D)** | **(E)** |
| **Supplementary Figure 3**. Experimental effective attenuation coefficients (*μ_eff_*) of reference (filled symbols-dashed lines) and 3D printed materials (open symbols-dotted lines) for DM at 31 kVp and: (A) Rh filtration with grid (w/ grid) and (C) without grid (w/o grid); (B) Ag filtration w/ grid and (D) w/o grid, and for DBT at 31 kVp and (E) Al filtration w/o grid. Error bars are not visible except for the thickness of 0.5 cm. (Lines are only for guidance). | | |

| 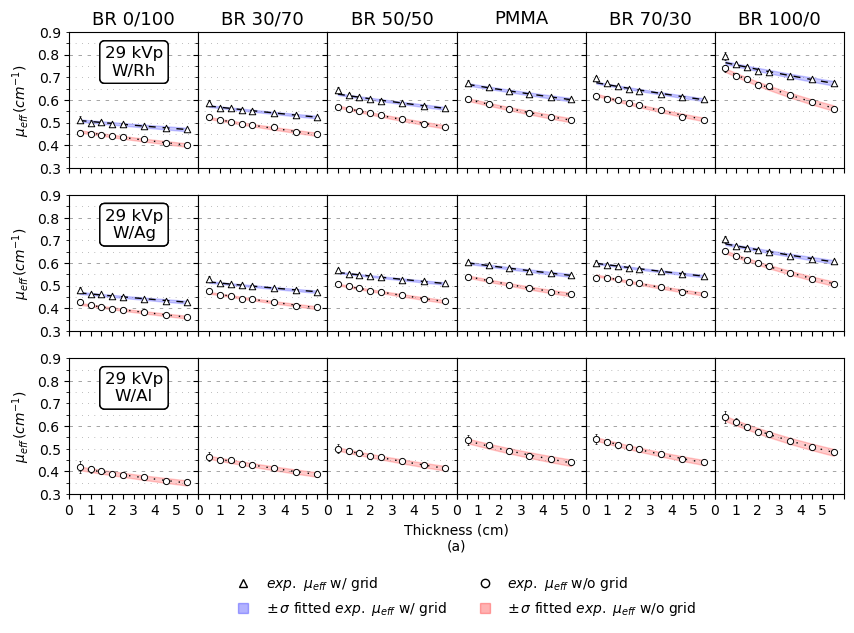 |
| --- |
| **(A)** |
| 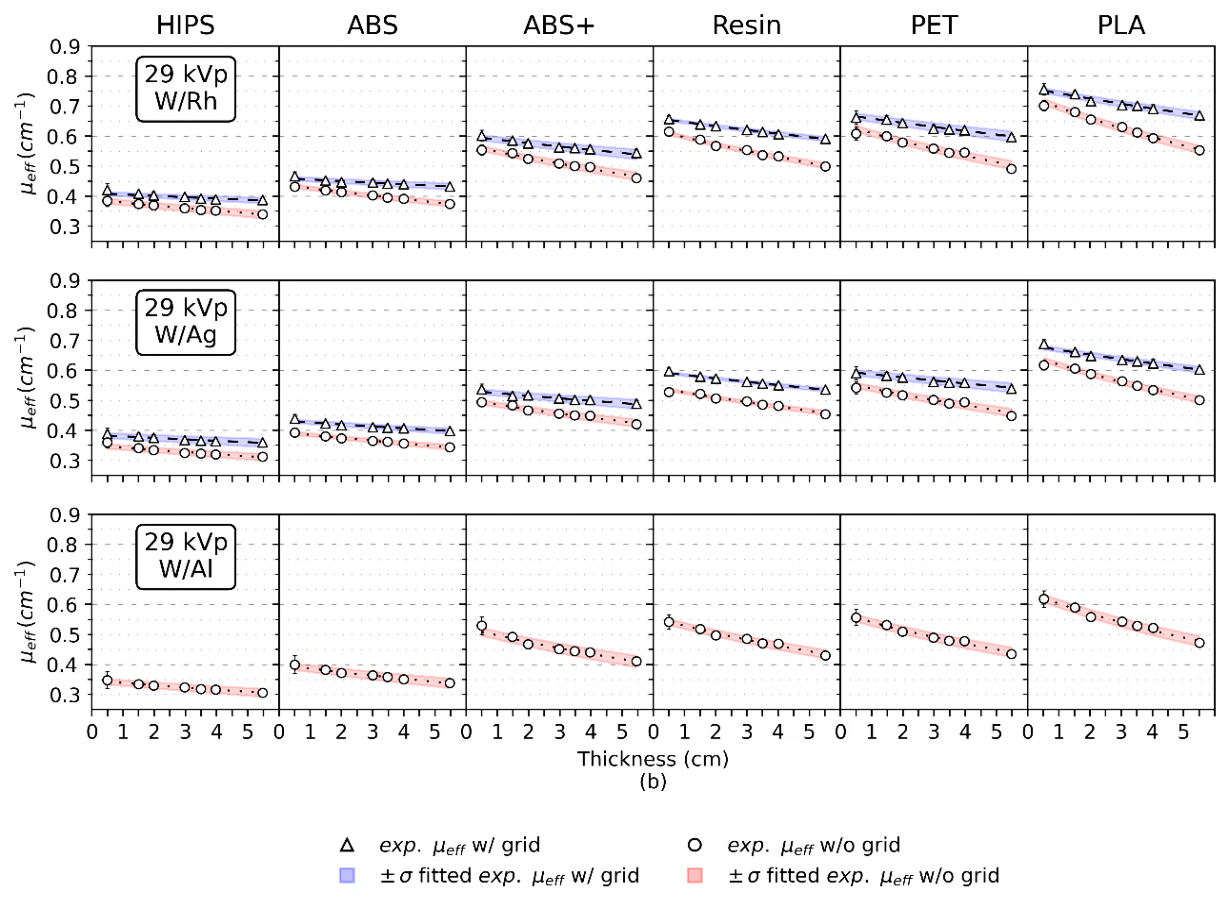 |
| **(B)** |
| **Supplementary Figure 4.** Weighted fittings provided by Equation 2 of experimental *μ_eff_* values obtained with the S1 system at 29 kVp with Rh, Ag and Al filtrations for **(A)** reference materials and **(B)** 3D-printed materials. 1-sigma bands of the fittings are in blue and pink for the experimental values with and without grid, respectively. |

## Supplementary Tables

| **Supplementary Table 1.** The *μ_0_* (cm^-1^) and *k* (x 10^-2^ cm^-1^) parameter values obtained from the fits of experimental *μ_eff_* values of reference materials to Equation 2 model and their uncertainties considering 1-sigma bands. | | | | | | | |
| --- | --- | --- | --- | --- | --- | --- | --- |
| ***μ_0_ (cm^-1^)* for reference materials** | | | | | | | |
| **Filter** | **kVp** | **BR 0/100** | **BR 30/70** | **BR 50/50** | **PMMA** | **BR 70/30** | **BR 100/0** |
| **Rh**  **w/**  **grid** | **27** | 0.532 ± 0.003 | 0.605 ± 0.004 | 0.664 ± 0.005 | 0.717 ± 0.005 | 0.719 ± 0.004 | 0.805 ± 0.004 |
|  | **29** | 0.514 ± 0.005 | 0.579 ± 0.004 | 0.633 ± 0.004 | 0.677 ± 0.005 | 0.685 ± 0.004 | 0.775 ± 0.006 |
|  | **31** | 0.499 ± 0.003 | 0.562 ± 0.004 | 0.610 ± 0.004 | 0.661 ± 0.004 | 0.658 ± 0.004 | 0.753 ± 0.004 |
| **Ag**  **w/**  **grid** | **27** | 0.488 ± 0.003 | 0.547 ± 0.004 | 0.595 ± 0.004 | 0.640 ± 0.004 | 0.641 ± 0.003 | 0.723 ± 0.004 |
|  | **29** | 0.473 ± 0.004 | 0.521 ± 0.003 | 0.564 ± 0.003 | 0.607 ± 0.004 | 0.605 ± 0.003 | 0.694 ± 0.005 |
|  | **31** | 0.456 ± 0.003 | 0.509 ± 0.003 | 0.553 ± 0.004 | 0.594 ± 0.004 | 0.596 ± 0.003 | 0.675 ± 0.004 |
| **Al**  **w/o**  **grid** | **27** | 0.456 ± 0.007 | 0.515 ± 0.009 | 0.565 ± 0.009 | 0.59 ± 0.01 | 0.615 ± 0.009 | 0.703 ± 0.010 |
|  | **29** | 0.421 ± 0.008 | 0.472 ± 0.007 | 0.509 ± 0.007 | 0.55 ± 0.01 | 0.556 ± 0.008 | 0.65 ± 0.01 |
|  | **31** | 0.390 ± 0.006 | 0.428 ± 0.007 | 0.465 ± 0.007 | 0.486 ± 0.008 | 0.501 ± 0.006 | 0.576 ± 0.007 |
| **Rh**  **w/o**  **grid** | **27** | 0.485 ± 0.005 | 0.557 ± 0.004 | 0.612 ± 0.005 | 0.656 ± 0.006 | 0.680 ± 0.005 | 0.778 ± 0.006 |
|  | **29** | 0.468 ± 0.003 | 0.529 ± 0.004 | 0.582 ± 0.005 | 0.614 ± 0.006 | 0.636 ± 0.005 | 0.754 ± 0.008 |
|  | **31** | 0.446 ± 0.003 | 0.501 ± 0.004 | 0.558 ± 0.005 | 0.597 ± 0.005 | 0.613 ± 0.004 | 0.714 ± 0.005 |
| **Ag**  **w/o**  **grid** | **27** | 0.434 ± 0.004 | 0.497 ± 0.004 | 0.546 ± 0.005 | 0.577 ± 0.005 | 0.591 ± 0.004 | 0.680 ± 0.005 |
|  | **29** | 0.427 ± 0.004 | 0.477 ± 0.004 | 0.514 ± 0.004 | 0.551 ± 0.005 | 0.556 ± 0.004 | 0.667 ± 0.006 |
|  | **31** | 0.399 ± 0.003 | 0.454 ± 0.004 | 0.495 ± 0.004 | 0.528 ± 0.004 | 0.539 ± 0.004 | 0.622 ± 0.004 |
| ***k (x 10^-2^ cm^-1^)* for reference materials** | | | | | | | |
| **Rh**  **w/**  **grid** | **27** | 1.7 ± 0.2 | 2.1 ± 0.2 | 2.6 ± 0.2 | 2.8 ± 0.2 | 2.8 ± 0.2 | 2.8 ± 0.2 |
|  | **29** | 1.7 ± 0.3 | 1.9 ± 0.2 | 2.3 ± 0.2 | 2.4 ± 0.2 | 2.5 ± 0.2 | 2.7 ± 0.3 |
|  | **31** | 1.8 ± 0.2 | 2.1 ± 0.2 | 2.3 ± 0.2 | 2.7 ± 0.2 | 2.7 ± 0.2 | 3.3 ± 0.2 |
| **Ag**  **w/**  **grid** | **27** | 1.8 ± 0.2 | 2.0 ± 0.2 | 2.4 ± 0.2 | 2.5 ± 0.2 | 2.5 ± 0.2 | 2.8 ± 0.2 |
|  | **29** | 1.9 ± 0.2 | 1.8 ± 0.2 | 1.9 ± 0.2 | 2.1 ± 0.2 | 2.1 ± 0.2 | 2.6 ± 0.2 |
|  | **31** | 1.8 ± 0.2 | 1.9 ± 0.2 | 2.2 ± 0.2 | 2.3 ± 0.2 | 2.5 ± 0.2 | 2.8 ± 0.2 |
| **Al**  **w/o**  **grid** | **27** | 3.6 ± 0.5 | 4.0 ± 0.6 | 4.6 ± 0.5 | 4.6 ± 0.6 | 5.3 ± 0.5 | 5.7 ± 0.5 |
|  | **29** | 3.8 ± 0.6 | 4.0 ± 0.5 | 4.2 ± 0.5 | 4.7 ± 0.6 | 4.9 ± 0.5 | 6.3 ± 0.6 |
|  | **31** | 3.8 ± 0.5 | 3.7 ± 0.5 | 4.1 ± 0.5 | 4.1 ± 0.5 | 4.4 ± 0.4 | 5.3 ± 0.4 |
| **Rh**  **w/o**  **grid** | **27** | 3.1 ± 0.4 | 3.7 ± 0.5 | 4.3 ± 0.5 | 4.7 ± 0.6 | 5.2 ± 0.5 | 5.8 ± 0.6 |
|  | **29** | 3.1 ± 0.5 | 3.3 ± 0.4 | 3.8 ± 0.5 | 3.8 ± 0.6 | 4.3 ± 0.5 | 6.1 ± 0.8 |
|  | **31** | 2.8 ± 0.3 | 2.9 ± 0.4 | 3.8 ± 0.5 | 4.2 ± 0.5 | 4.6 ± 0.4 | 5.6 ± 0.5 |
| **Ag**  **w/o**  **grid** | **27** | 2.9 ± 0.3 | 3.4 ± 0.4 | 4.0 ± 0.5 | 4.1 ± 0.5 | 4.3 ± 0.4 | 5.0 ± 0.5 |
|  | **29** | 3.3 ± 0.4 | 3.3 ± 0.4 | 3.6 ± 0.4 | 3.7 ± 0.5 | 3.7 ± 0.4 | 5.6 ± 0.6 |
|  | **31** | 2.5 ± 0.3 | 2.9 ± 0.4 | 3.3 ± 0.4 | 3.6 ± 0.4 | 3.8 ± 0.4 | 4.6 ± 0.4 |

| **Supplementary Table 2.** The *μ_0_* (cm^-1^) and *k* (x 10^-2^ cm^-1^) parameter values obtained from the fits of experimental *μ_eff_* values of 3D printed materials to Equation 2 model and their uncertainties considering 1-sigma bands. | | | | | | | |
| --- | --- | --- | --- | --- | --- | --- | --- |
| ***μ_0_ (cm^-1^)* for 3D printed materials** | | | | | | | |
| **Filter** | **kVp** | **HIPS** | **ABS** | **ABS+** | **Resin** | **PET** | **PLA** |
| **Rh**  **w/**  **grid** | **27** | 0.428 ± 0.009 | 0.476 ± 0.005 | 0.630 ± 0.010 | 0.686 ± 0.004 | 0.70 ± 0.01 | 0.796 ± 0.007 |
|  | **29** | 0.410 ± 0.006 | 0.461 ± 0.006 | 0.601 ± 0.009 | 0.661 ± 0.005 | 0.67 ± 0.01 | 0.761 ± 0.008 |
|  | **31** | 0.404 ± 0.009 | 0.455 ± 0.005 | 0.590 ± 0.009 | 0.646 ± 0.004 | 0.66 ± 0.01 | 0.741 ± 0.007 |
| **Ag**  **w/**  **grid** | **27** | 0.398 ± 0.009 | 0.446 ± 0.005 | 0.560 ± 0.009 | 0.630 ± 0.003 | 0.63 ± 0.01 | 0.718 ± 0.006 |
|  | **29** | 0.385 ± 0.009 | 0.433 ± 0.006 | 0.533 ± 0.009 | 0.597 ± 0.004 | 0.598 ± 0.010 | 0.684 ± 0.007 |
|  | **31** | 0.375 ± 0.008 | 0.423 ± 0.005 | 0.531 ± 0.008 | 0.584 ± 0.003 | 0.588 ± 0.010 | 0.667 ± 0.006 |
| **Al**  **w/o**  **grid** | **27** | 0.37 ± 0.01 | 0.422 ± 0.009 | 0.56 ± 0.01 | 0.61 ± 0.01 | 0.62 ± 0.01 | 0.69 ± 0.01 |
|  | **29** | 0.347 ± 0.009 | 0.40 ± 0.01 | 0.52 ± 0.01 | 0.55 ± 0.01 | 0.57 ± 0.01 | 0.64 ± 0.01 |
|  | **31** | 0.334 ± 0.009 | 0.372 ± 0.008 | 0.46 ± 0.01 | 0.503 ± 0.008 | 0.510 ± 0.011 | 0.578 ± 0.010 |
| **Rh**  **w/o**  **grid** | **27** | 0.396 ± 0.009 | 0.444 ± 0.005 | 0.59 ± 0.009 | 0.642 ± 0.005 | 0.67 ± 0.01 | 0.76 ± 0.01 |
|  | **29** | 0.389 ± 0.009 | 0.44 ± 0.006 | 0.57 ± 0.008 | 0.622 ± 0.006 | 0.64 ± 0.01 | 0.74 ± 0.01 |
|  | **31** | 0.364 ± 0.008 | 0.414 ± 0.005 | 0.55 ± 0.008 | 0.591 ± 0.005 | 0.62 ± 0.01 | 0.699 ± 0.008 |
| **Ag**  **w/o**  **grid** | **27** | 0.358 ± 0.008 | 0.403 ± 0.005 | 0.527 ± 0.008 | 0.576 ± 0.005 | 0.59 ± 0.01 | 0.671 ± 0.007 |
|  | **29** | 0.35 ± 0.008 | 0.395 ± 0.005 | 0.501 ± 0.008 | 0.544 ± 0.005 | 0.561 ± 0.009 | 0.652 ± 0.008 |
|  | **31** | 0.335 ± 0.007 | 0.367 ± 0.004 | 0.488 ± 0.007 | 0.524 ± 0.004 | 0.546 ± 0.009 | 0.615 ± 0.007 |
| ***k (x 10^-2^ cm^-1^)* for 3D printed materials** | | | | | | | |
| **Rh**  **w/**  **grid** | **27** | 1.3 ± 0.6 | 1.3 ± 0.3 | 2.4 ± 0.6 | 2.4 ± 0.2 | 2.5 ± 0.5 | 2.7 ± 0.3 |
|  | **29** | 1.1 ± 0.4 | 1.2 ± 0.4 | 2.1 ± 0.5 | 2.2 ± 0.2 | 2.2 ± 0.5 | 2.5 ± 0.3 |
|  | **31** | 1.3 ± 0.6 | 1.6 ± 0.3 | 2.5 ± 0.5 | 2.6 ± 0.2 | 2.6 ± 0.5 | 3.0 ± 0.3 |
| **Ag**  **w/**  **grid** | **27** | 1.4 ± 0.6 | 1.7 ± 0.3 | 2.0 ± 0.6 | 2.6 ± 0.2 | 2.2 ± 0.5 | 2.8 ± 0.3 |
|  | **29** | 1.4 ± 0.6 | 1.6 ± 0.4 | 1.7 ± 0.6 | 2.1 ± 0.2 | 1.9 ± 0.5 | 2.5 ± 0.3 |
|  | **31** | 1.4 ± 0.6 | 1.7 ± 0.3 | 2.3 ± 0.6 | 2.3 ± 0.2 | 2.1 ± 0.5 | 2.6 ± 0.2 |
| **Al**  **w/o**  **grid** | **27** | 2.5 ± 0.8 | 3.3 ± 0.6 | 4.7 ± 0.9 | 5.5 ± 0.6 | 5.5 ± 0.8 | 5.8 ± 0.7 |
|  | **29** | 2.4 ± 0.7 | 3.3 ± 0.8 | 5.0 ± 0.9 | 5.0 ± 0.6 | 5.3 ± 0.7 | 6.2 ± 0.7 |
|  | **31** | 2.9 ± 0.8 | 3.2 ± 0.6 | 4.0 ± 0.9 | 4.3 ± 0.5 | 4.4 ± 0.7 | 5.5 ± 0.6 |
| **Rh**  **w/o**  **grid** | **27** | 2.7 ± 0.6 | 3.0 ± 0.4 | 4.2 ± 0.6 | 4.3 ± 0.3 | 5.4 ± 0.6 | 5.5 ± 0.4 |
|  | **29** | 2.7 ± 0.6 | 3.2 ± 0.4 | 4.1 ± 0.6 | 4.3 ± 0.4 | 4.9 ± 0.6 | 5.9 ± 0.5 |
|  | **31** | 2.1 ± 0.6 | 2.6 ± 0.4 | 4.0 ± 0.6 | 4.0 ± 0.3 | 5.0 ± 0.6 | 5.6 ± 0.4 |
| **Ag**  **w/o**  **grid** | **27** | 2.2 ± 0.6 | 2.6 ± 0.4 | 3.7 ± 0.6 | 4.1 ± 0.3 | 4.6 ± 0.5 | 5.1 ± 0.4 |
|  | **29** | 2.3 ± 0.6 | 2.7 ± 0.4 | 3.3 ± 0.6 | 3.4 ± 0.3 | 4.0 ± 0.5 | 5.4 ± 0.4 |
|  | **31** | 1.9 ± 0.6 | 1.8 ± 0.3 | 3.4 ± 0.6 | 3.2 ± 0.3 | 4.0 ± 0.5 | 4.6 ± 0.4 |
